# Supplementary figures and images for: Particle morphology, structure and properties of nascent ultra-high molecular weight polyethylene
Source: R Soc Open Sci. 2020 Aug 5;7(8):200663. doi: 10.1098/rsos.200663 (PMC7481705; doi:10.1098/rsos.200663)

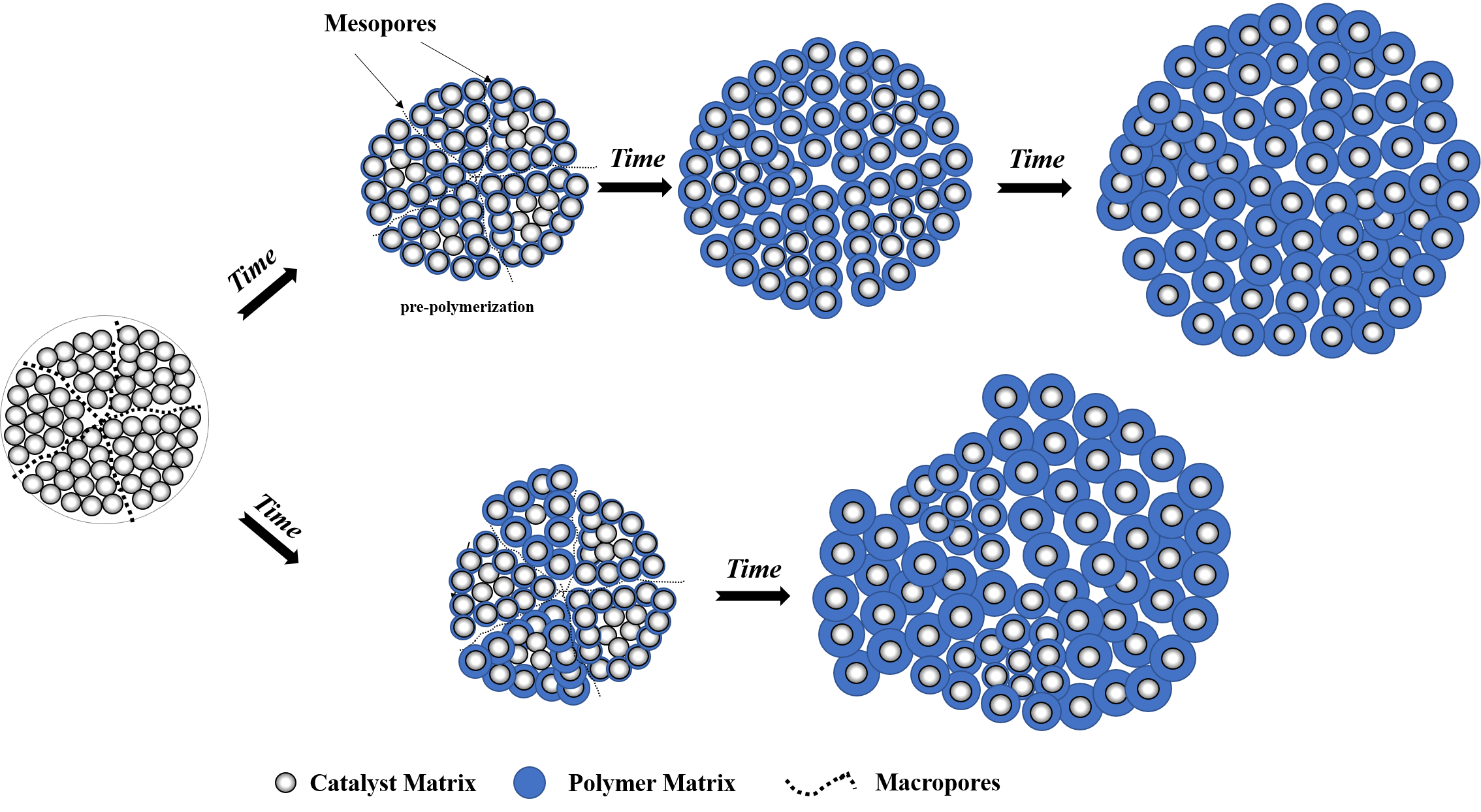

Supplement: The mechanism of UHMWPE particle growth [file rsos200663supp2.zip › Figure 2 The schematic diagrams on the mechanism of UHMWPE particle growth.tif]

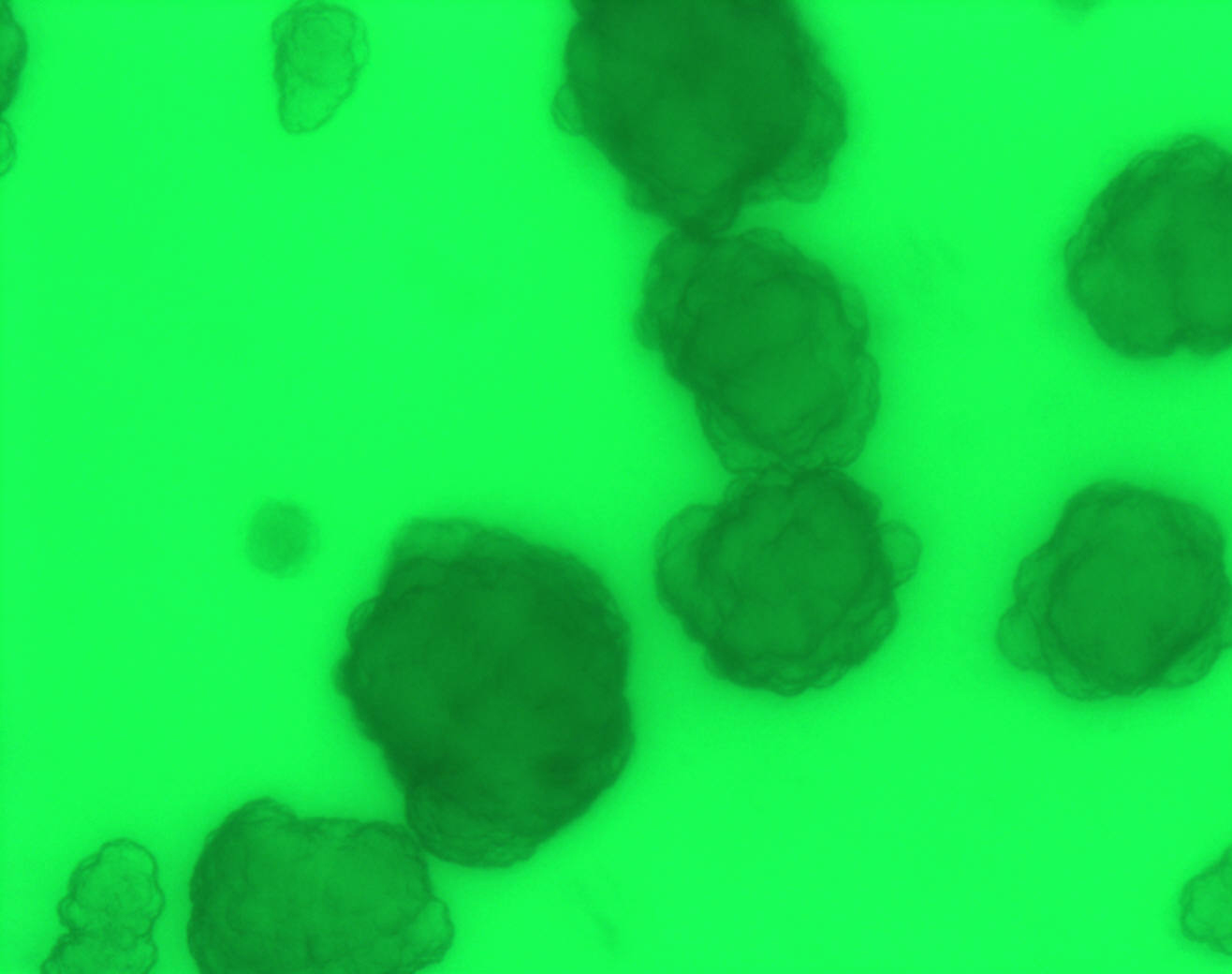

Supplement: Optical photographs [file rsos200663supp4.zip › Figure 4 Optical photographs of PE-140 in LP at 110íμ.jpg]

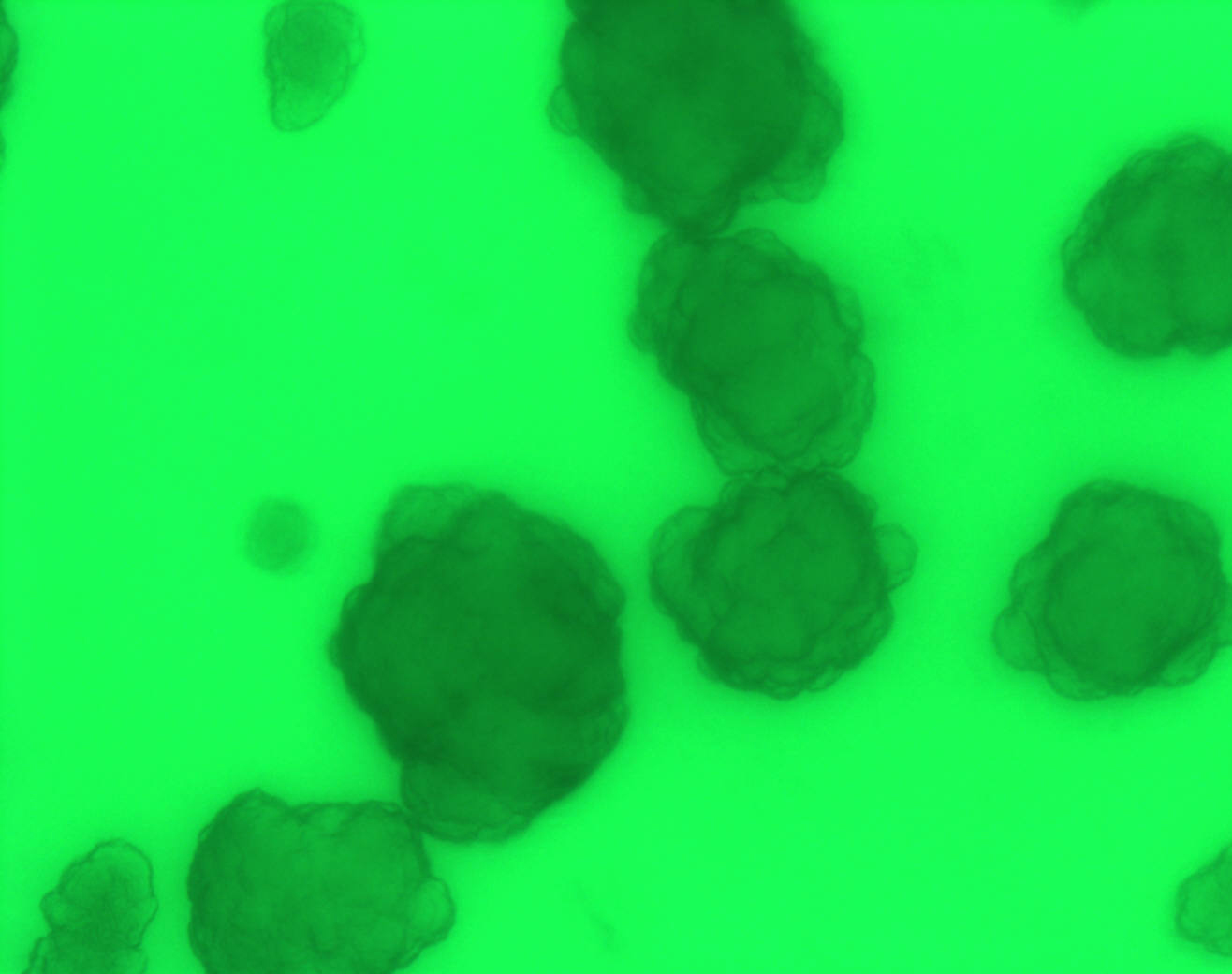

Supplement: Optical photographs [file rsos200663supp4.zip › Figure 4 Optical photographs of PE-140 in LP at 130íμ.jpg]

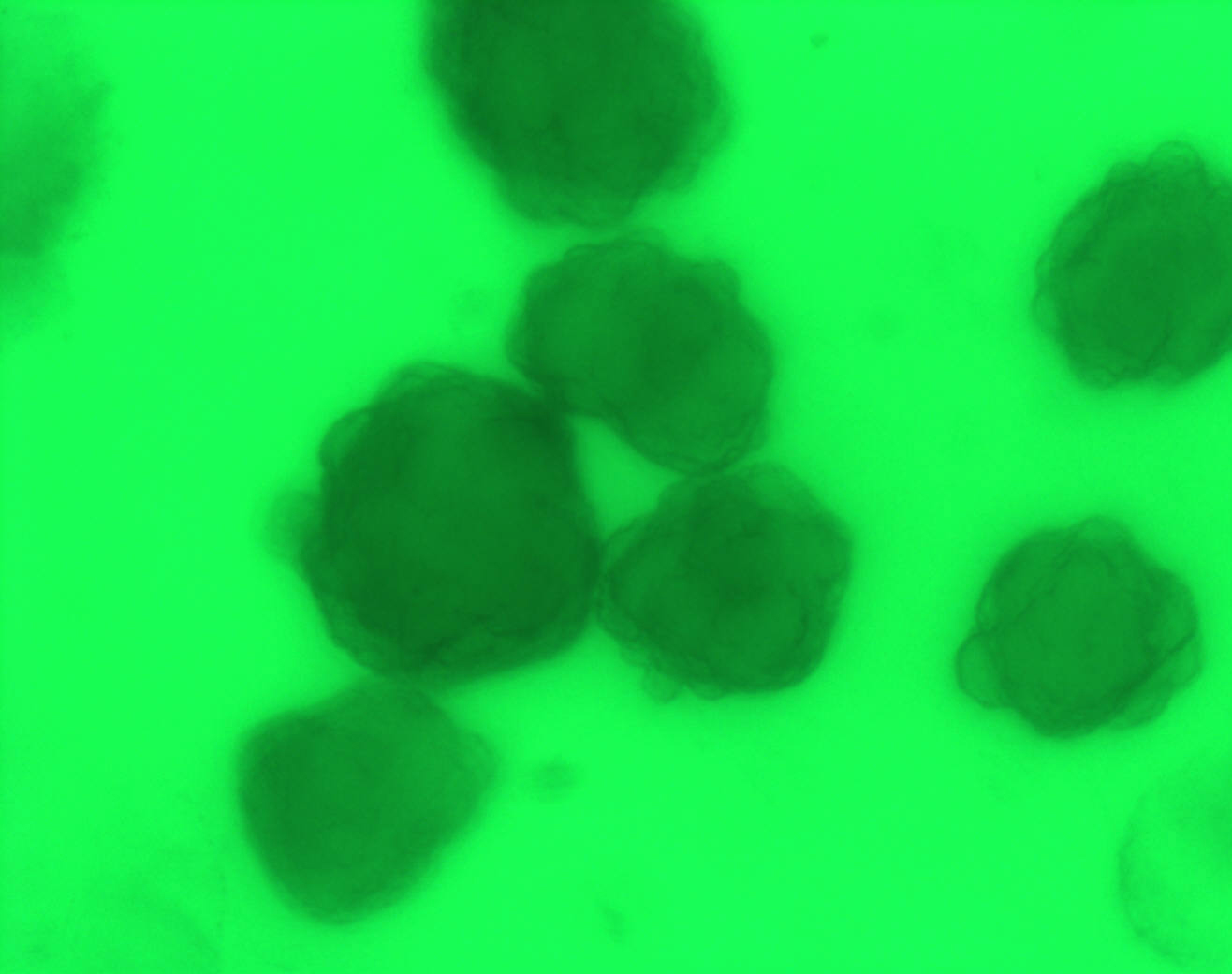

Supplement: Optical photographs [file rsos200663supp4.zip › Figure 4 Optical photographs of PE-140 in LP at 134.5íμ.jpg]

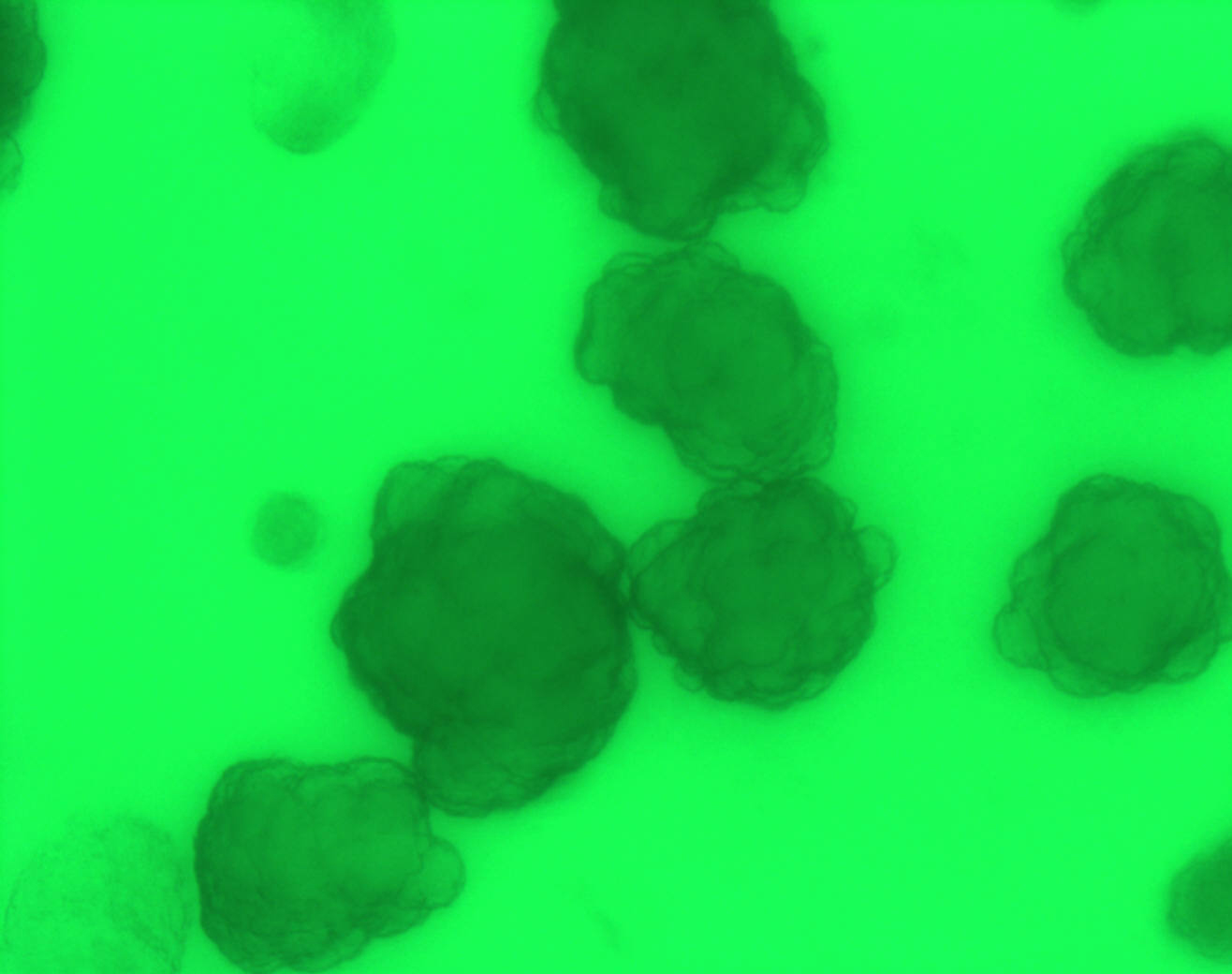

Supplement: Optical photographs [file rsos200663supp4.zip › Figure 4 Optical photographs of PE-140 in LP at 134íμ.jpg]

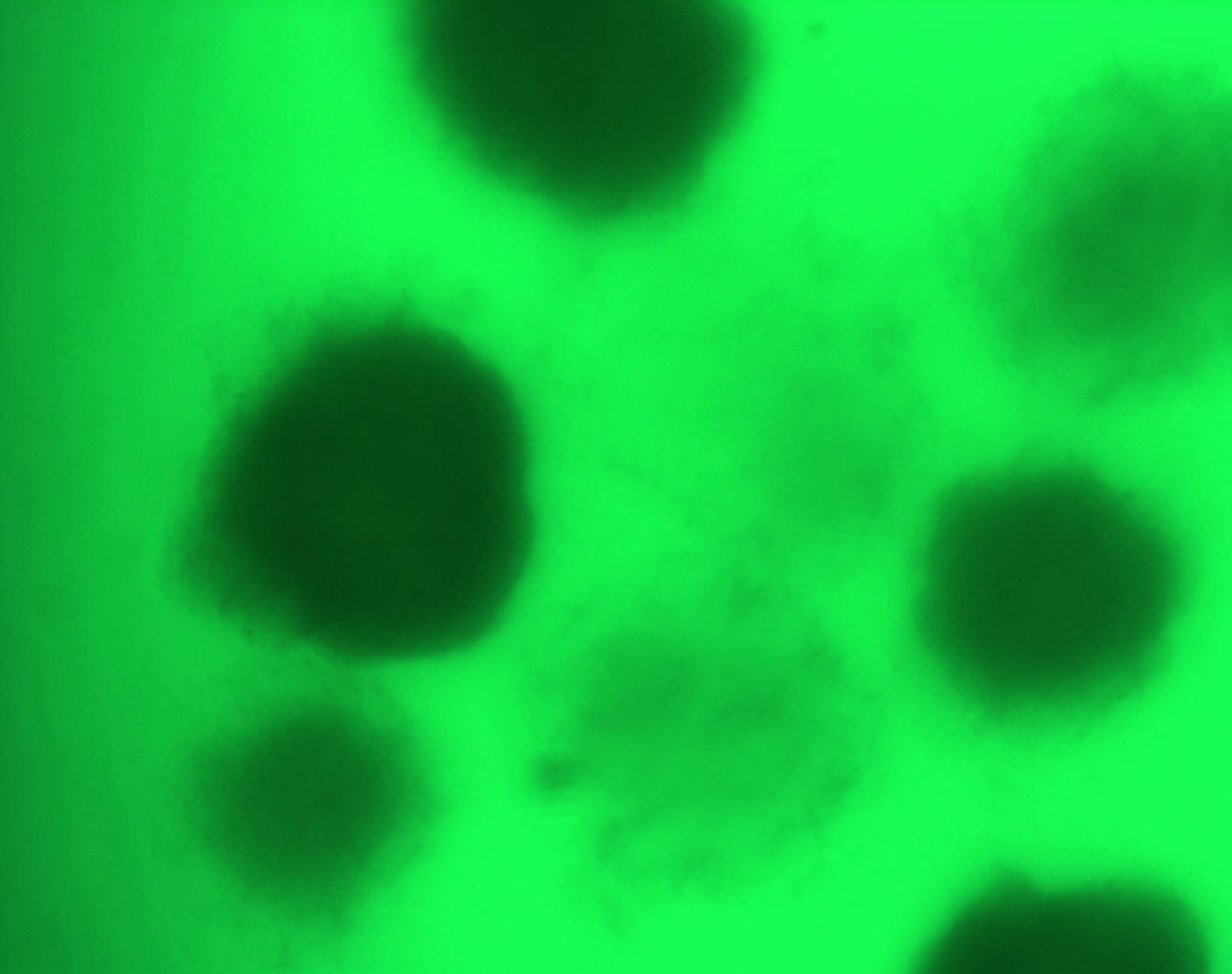

Supplement: Optical photographs [file rsos200663supp4.zip › Figure 4 Optical photographs of PE-140 in LP at 135.5íμ.jpg]

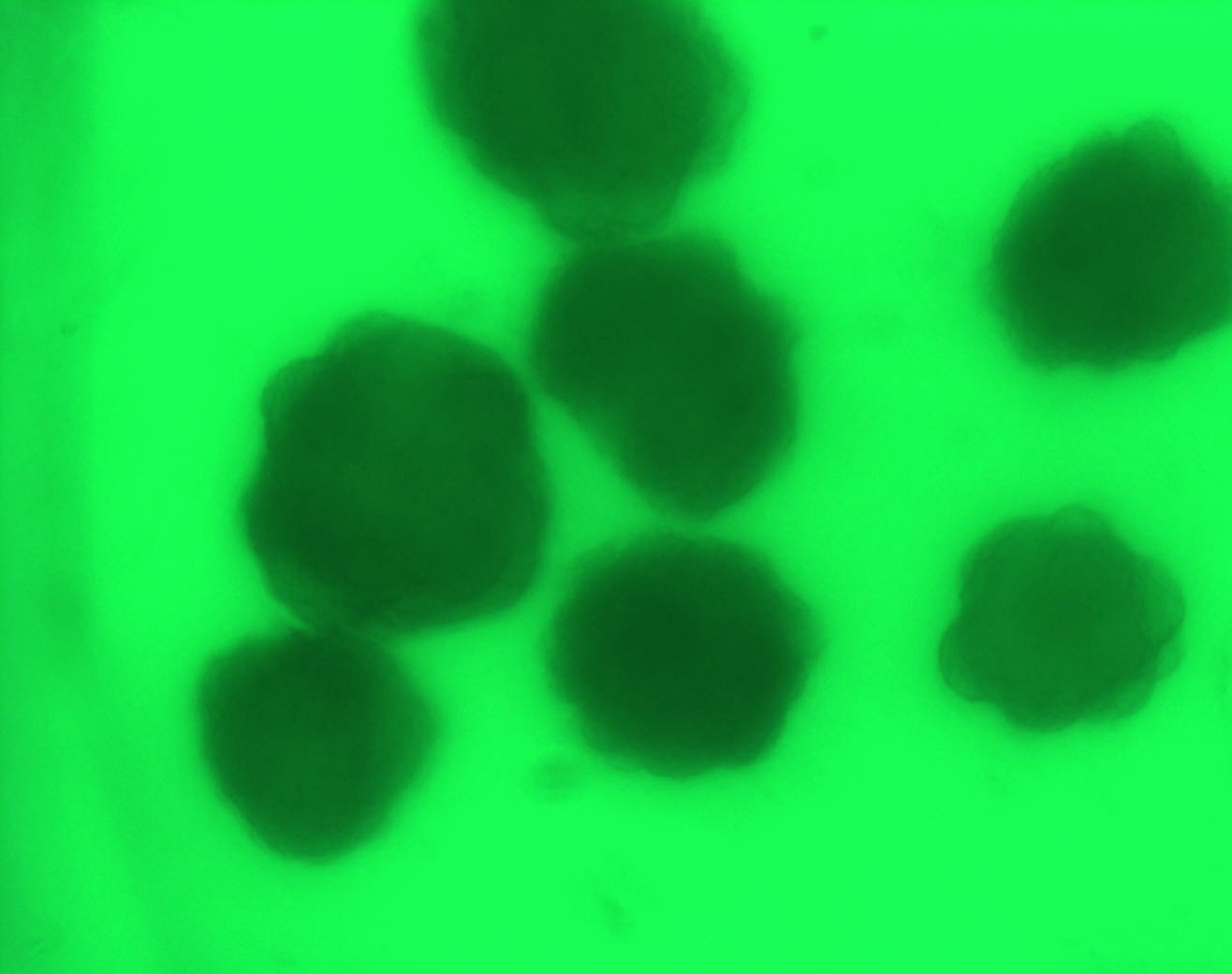

Supplement: Optical photographs [file rsos200663supp4.zip › Figure 4 Optical photographs of PE-140 in LP at 135íμ.jpg]

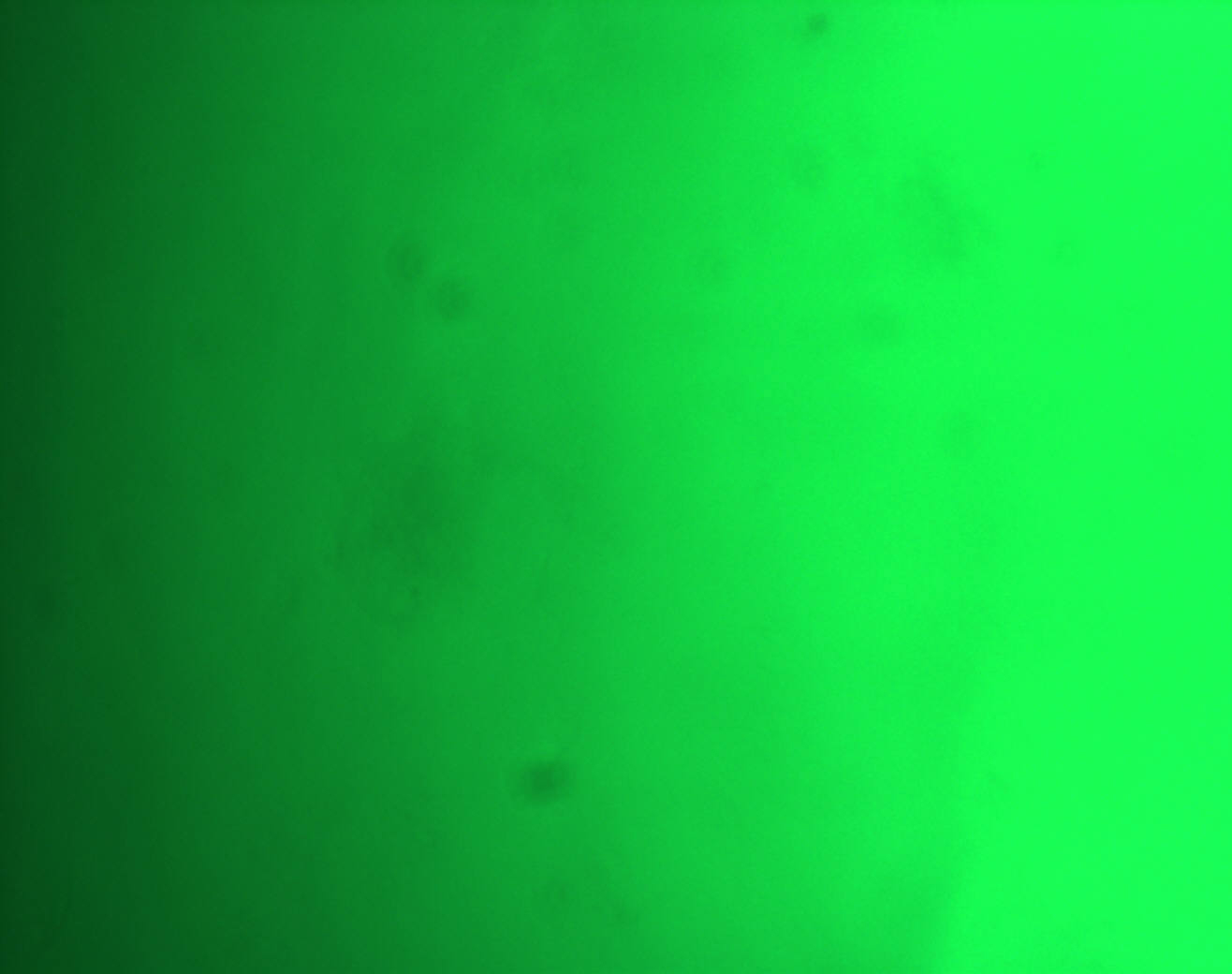

Supplement: Optical photographs [file rsos200663supp4.zip › Figure 4 Optical photographs of PE-140 in LP at 136.5íμ.jpg]

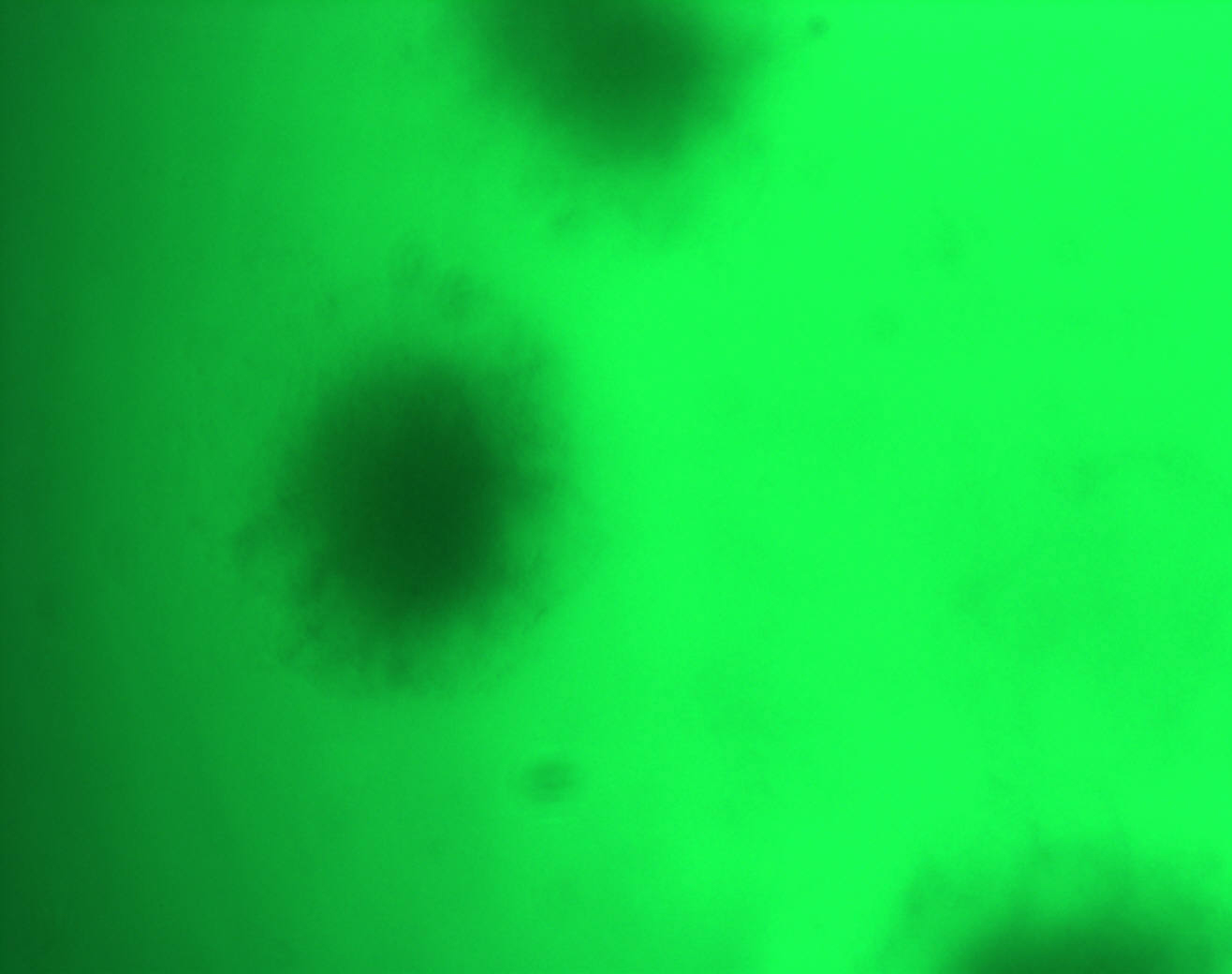

Supplement: Optical photographs [file rsos200663supp4.zip › Figure 4 Optical photographs of PE-140 in LP at 136íμ.jpg]

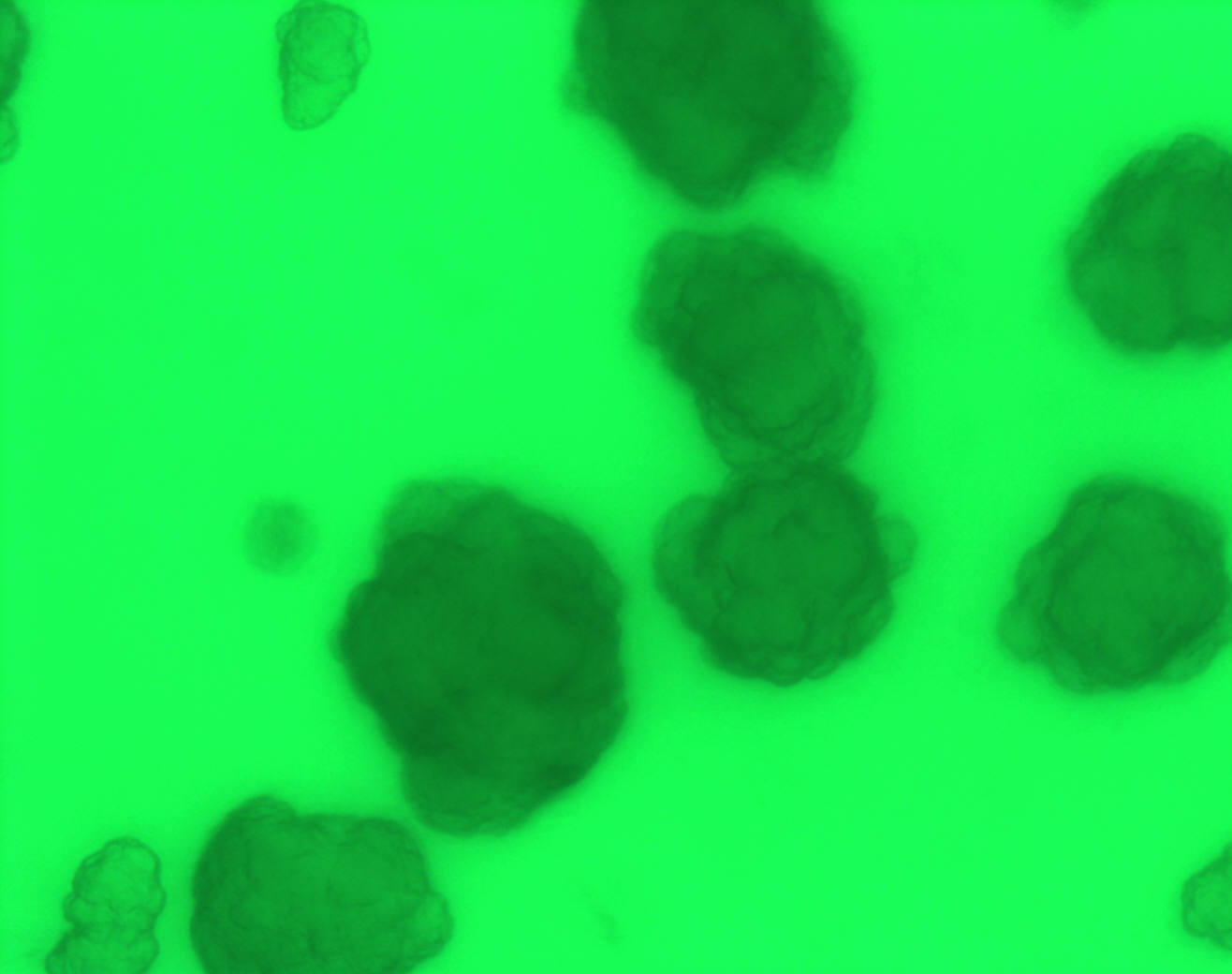

Supplement: Optical photographs [file rsos200663supp4.zip › Figure 4 Optical photographs of PE-140 in LP at 30íμ.jpg]

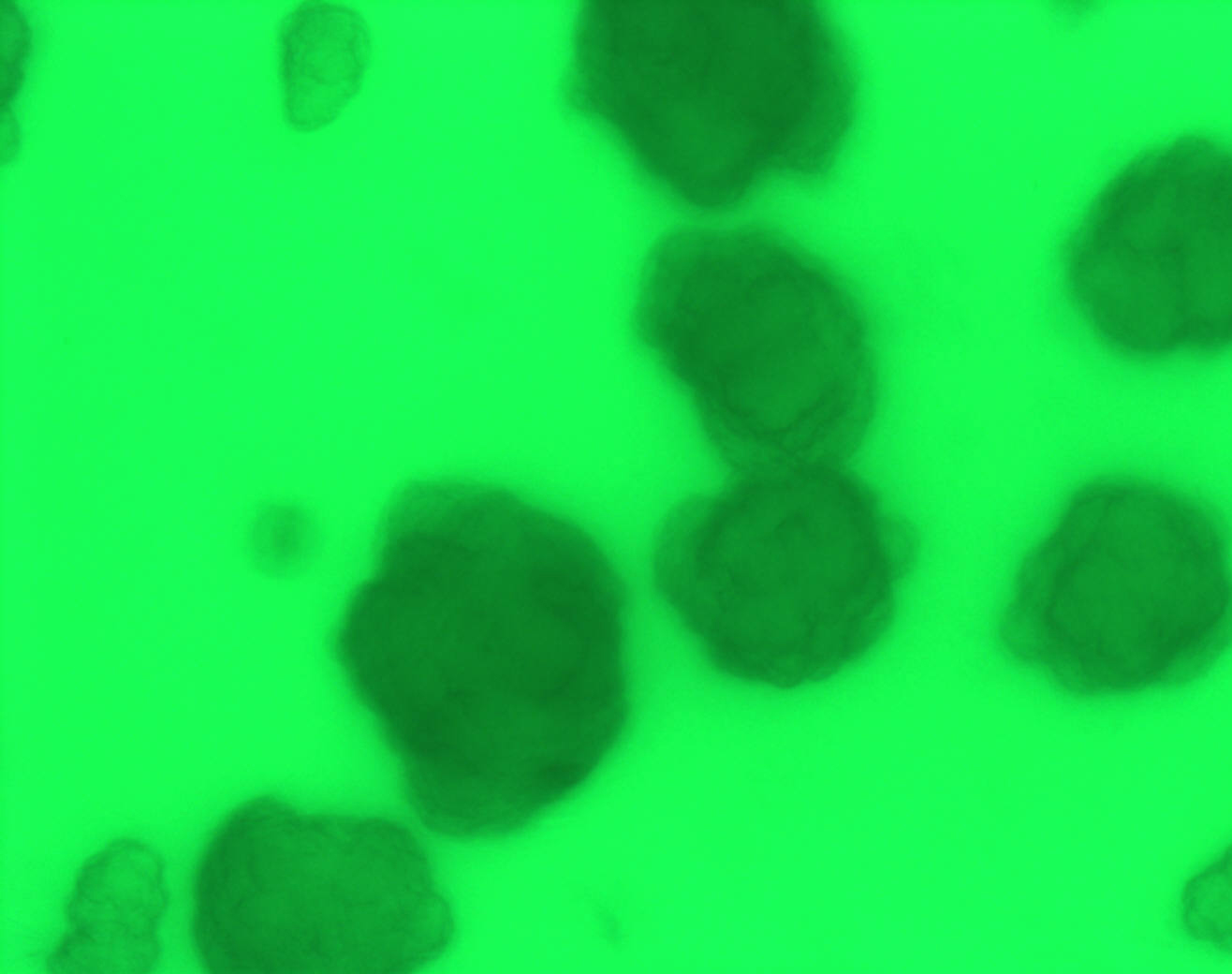

Supplement: Optical photographs [file rsos200663supp4.zip › Figure 4 Optical photographs of PE-140 in LP at 50íμ.jpg]

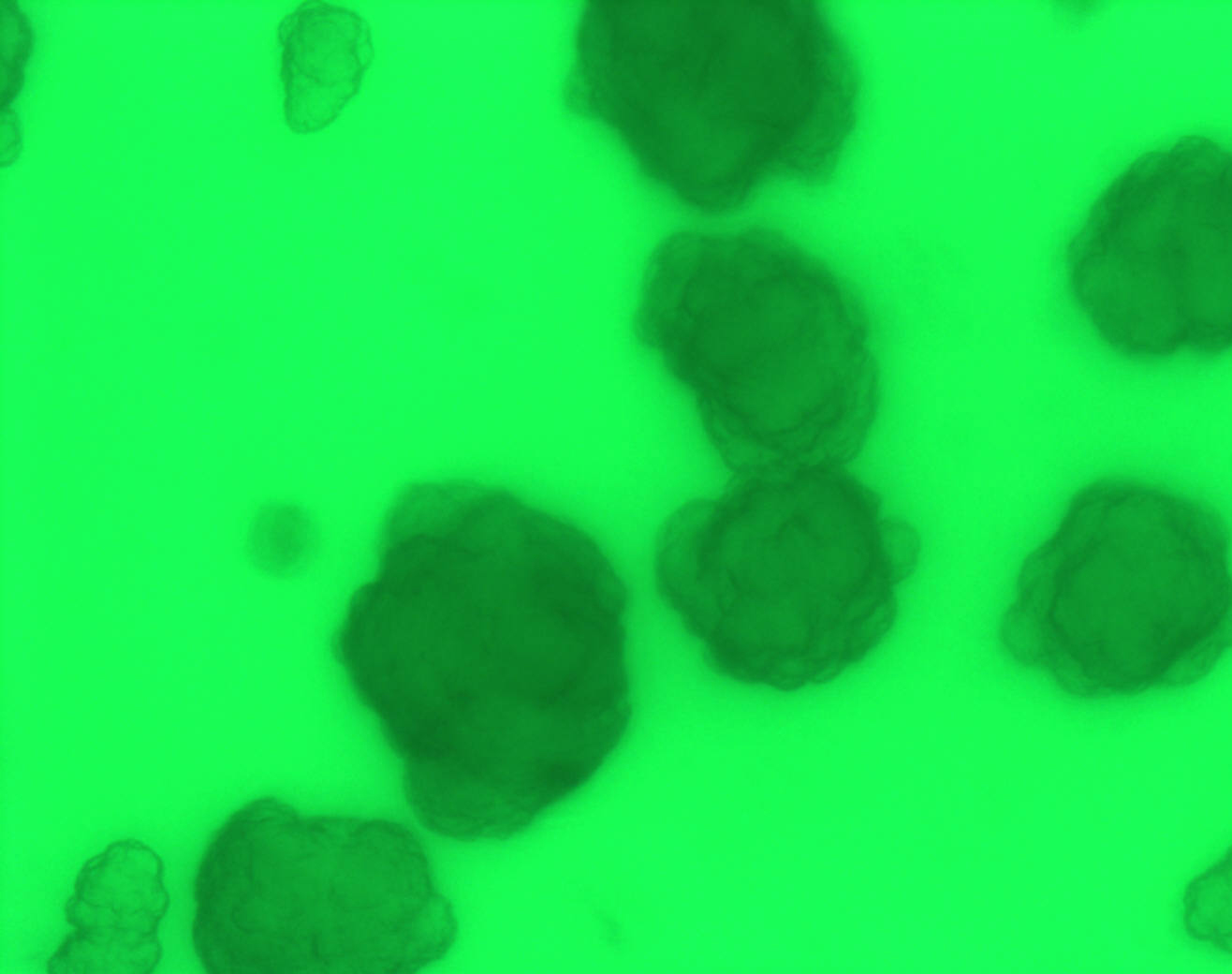

Supplement: Optical photographs [file rsos200663supp4.zip › Figure 4 Optical photographs of PE-140 in LP at 70íμ.jpg]

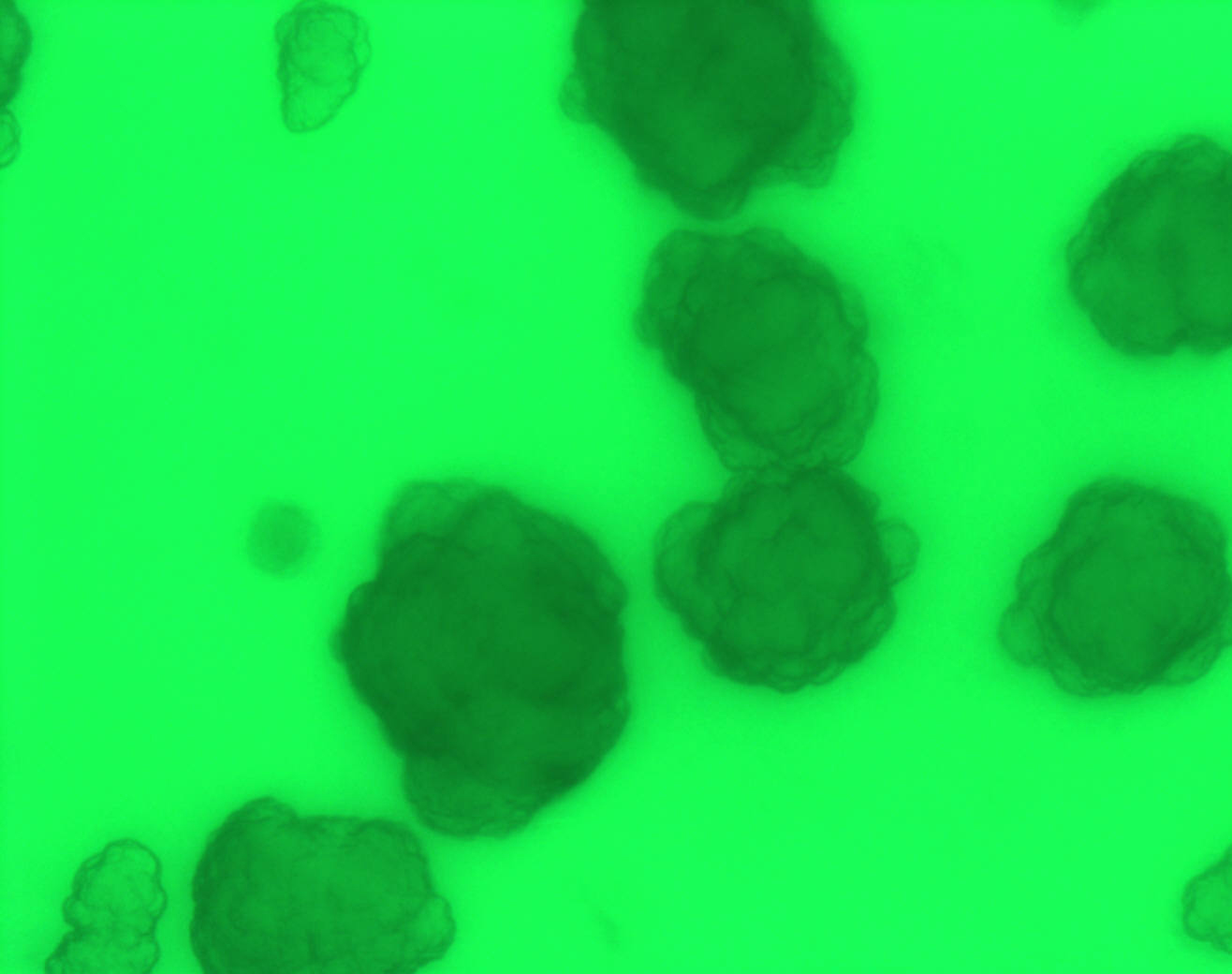

Supplement: Optical photographs [file rsos200663supp4.zip › Figure 4 Optical photographs of PE-140 in LP at 90íμ.jpg]

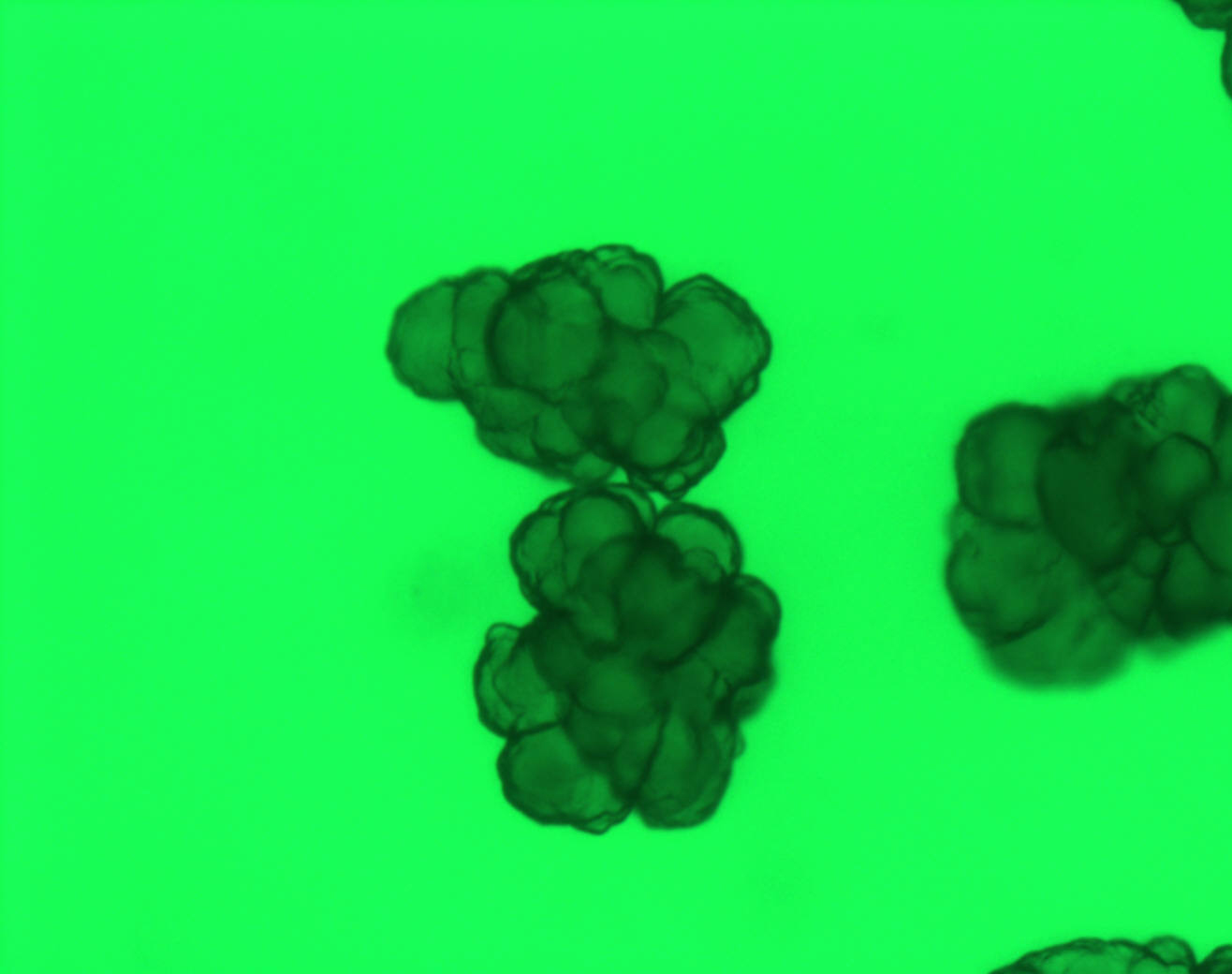

Supplement: Optical photographs [file rsos200663supp4.zip › Figure 5 Optical photographs of PE-120 in LP at 110íμ.jpg]

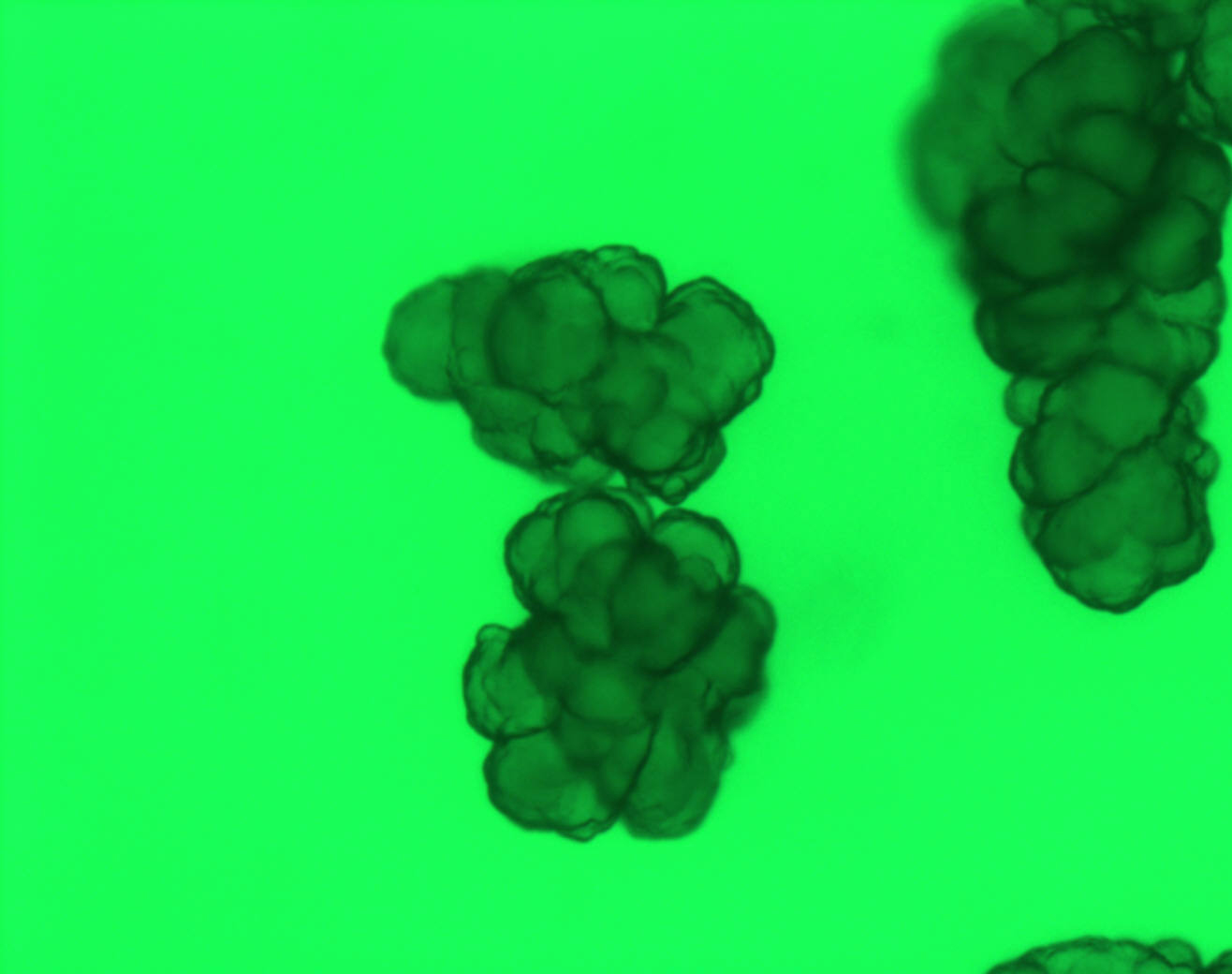

Supplement: Optical photographs [file rsos200663supp4.zip › Figure 5 Optical photographs of PE-120 in LP at 130íμ.jpg]

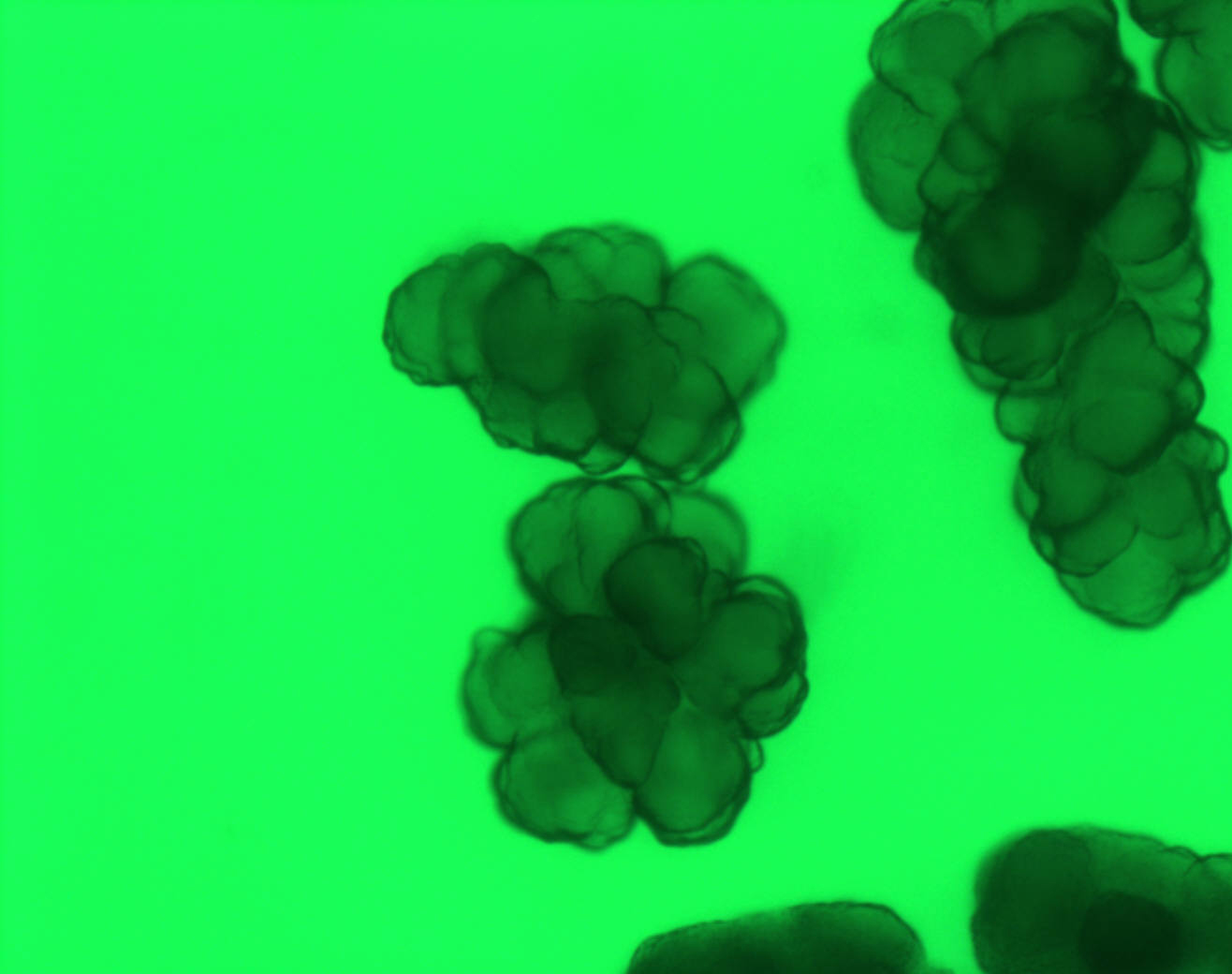

Supplement: Optical photographs [file rsos200663supp4.zip › Figure 5 Optical photographs of PE-120 in LP at 134.5íμ.jpg]

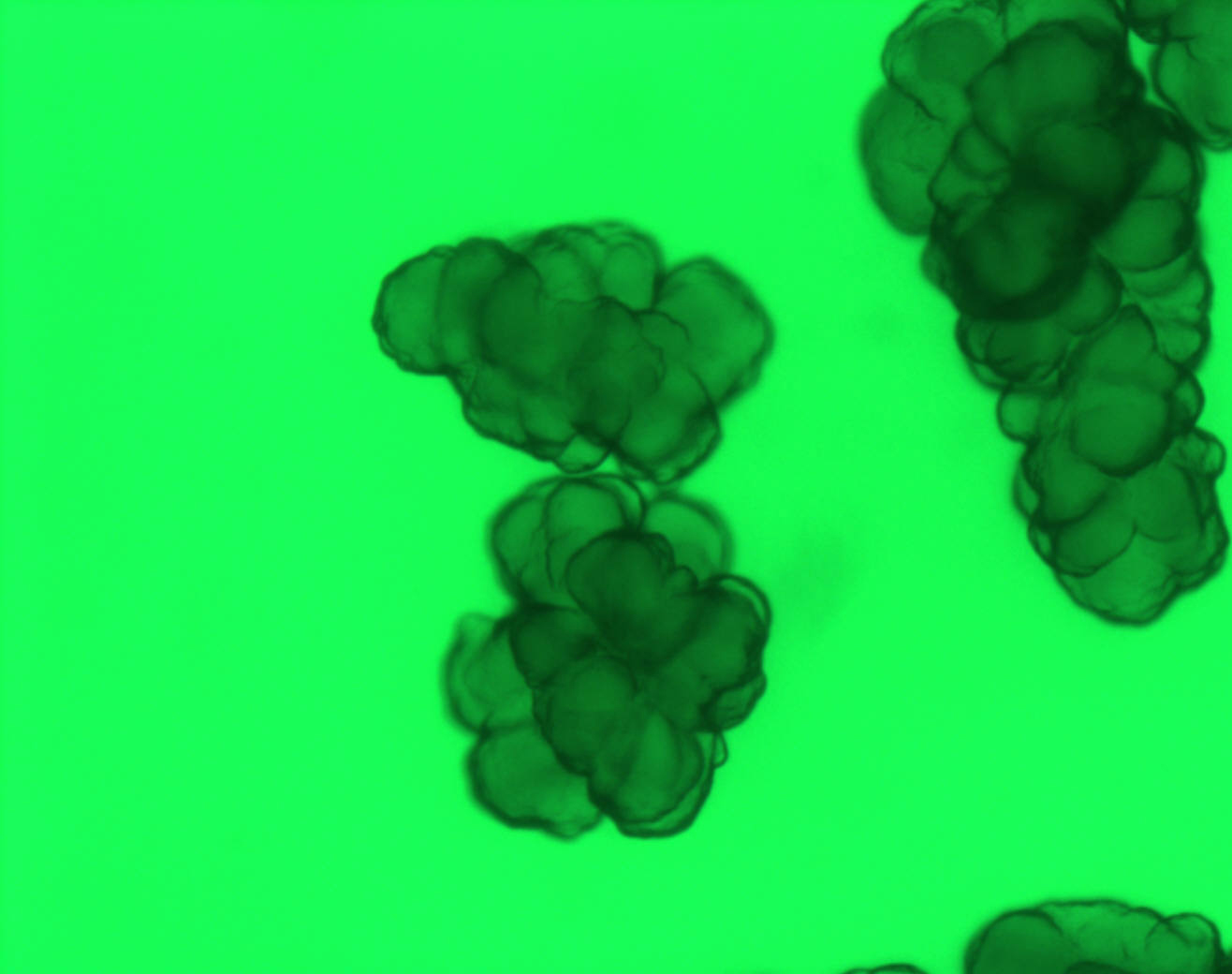

Supplement: Optical photographs [file rsos200663supp4.zip › Figure 5 Optical photographs of PE-120 in LP at 134íμ.jpg]

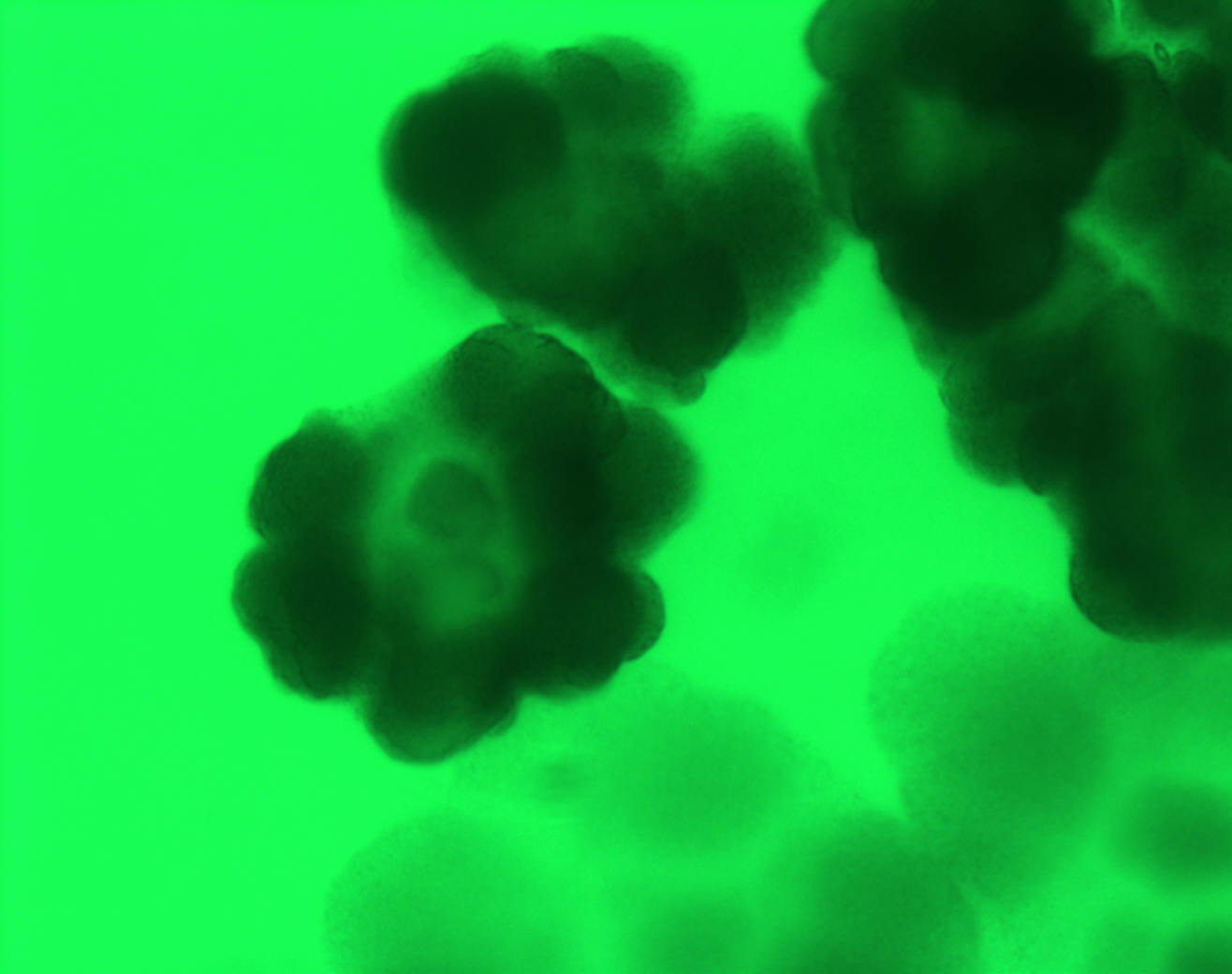

Supplement: Optical photographs [file rsos200663supp4.zip › Figure 5 Optical photographs of PE-120 in LP at 135.5íμ.jpg]

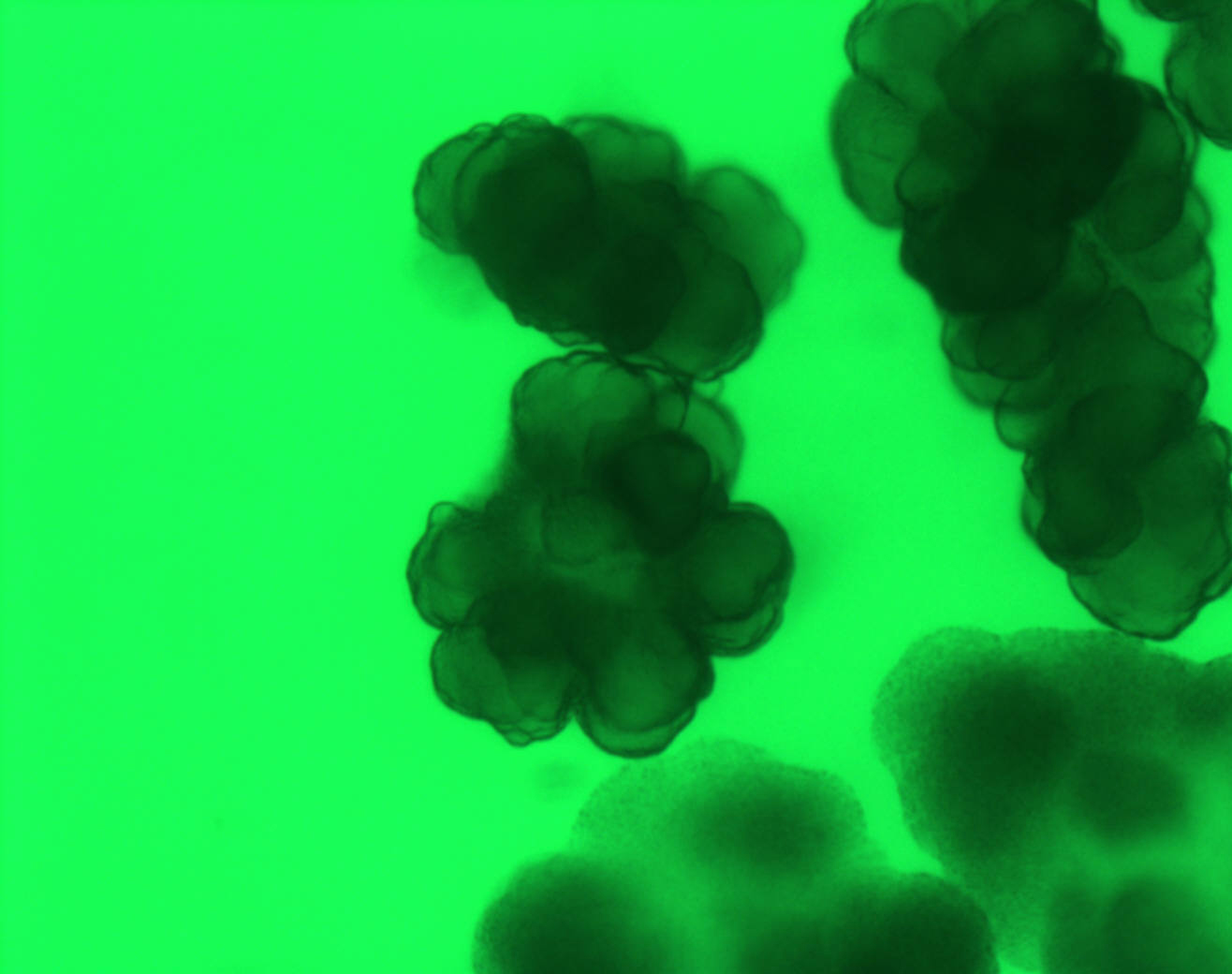

Supplement: Optical photographs [file rsos200663supp4.zip › Figure 5 Optical photographs of PE-120 in LP at 135íμ.jpg]

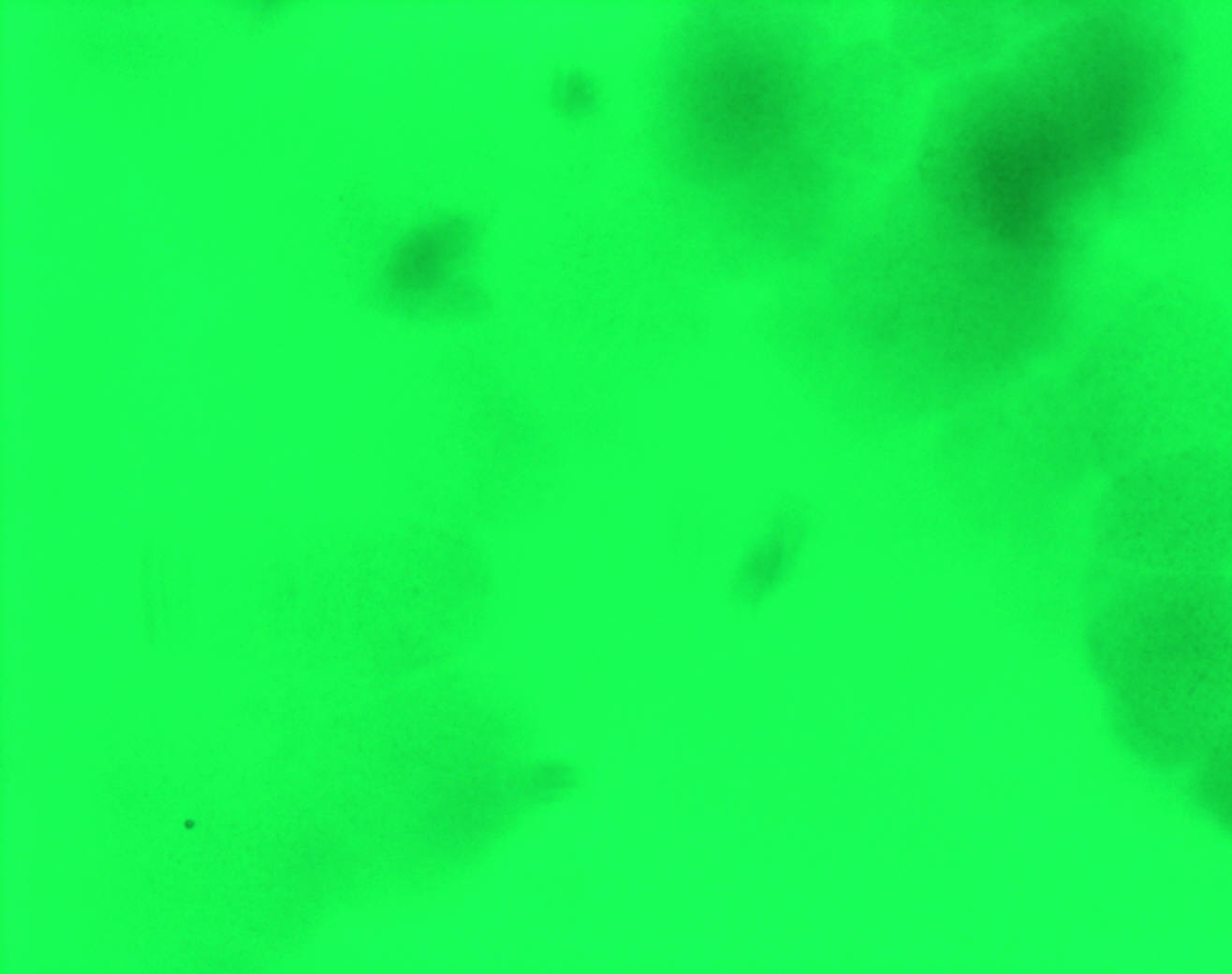

Supplement: Optical photographs [file rsos200663supp4.zip › Figure 5 Optical photographs of PE-120 in LP at 136.5íμ.jpg]

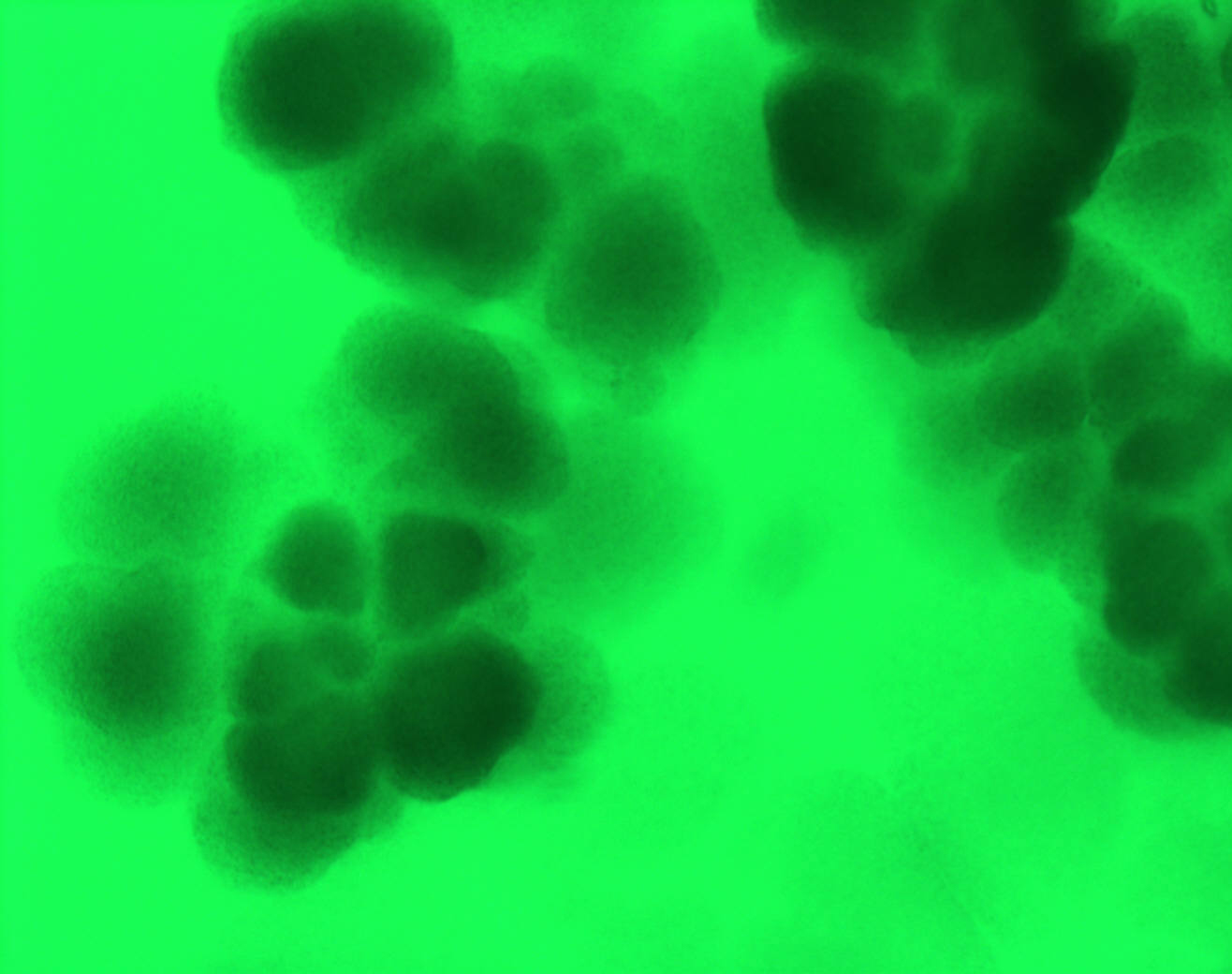

Supplement: Optical photographs [file rsos200663supp4.zip › Figure 5 Optical photographs of PE-120 in LP at 136íμ.jpg]

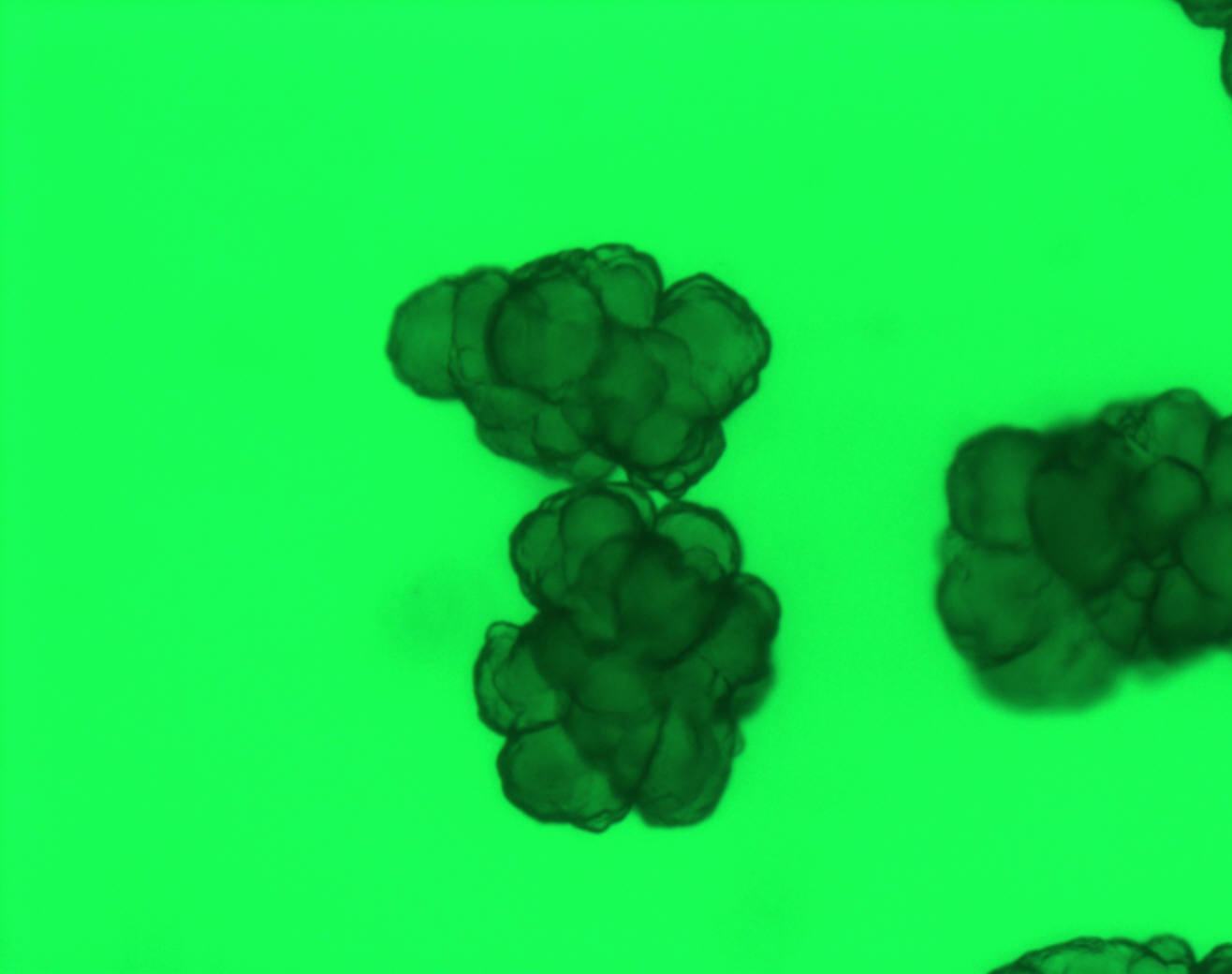

Supplement: Optical photographs [file rsos200663supp4.zip › Figure 5 Optical photographs of PE-120 in LP at 30íμ.jpg]

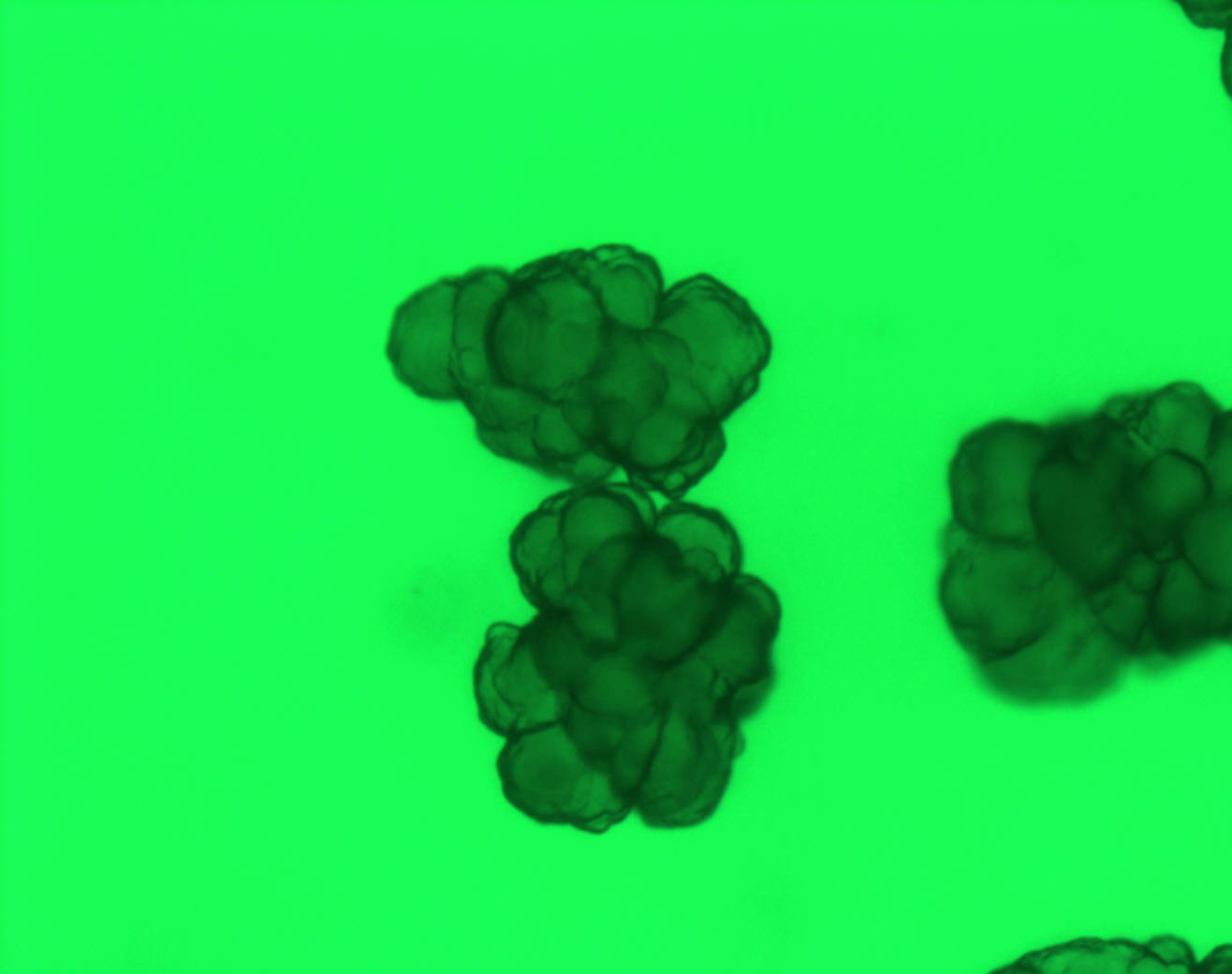

Supplement: Optical photographs [file rsos200663supp4.zip › Figure 5 Optical photographs of PE-120 in LP at 50íμ.jpg]

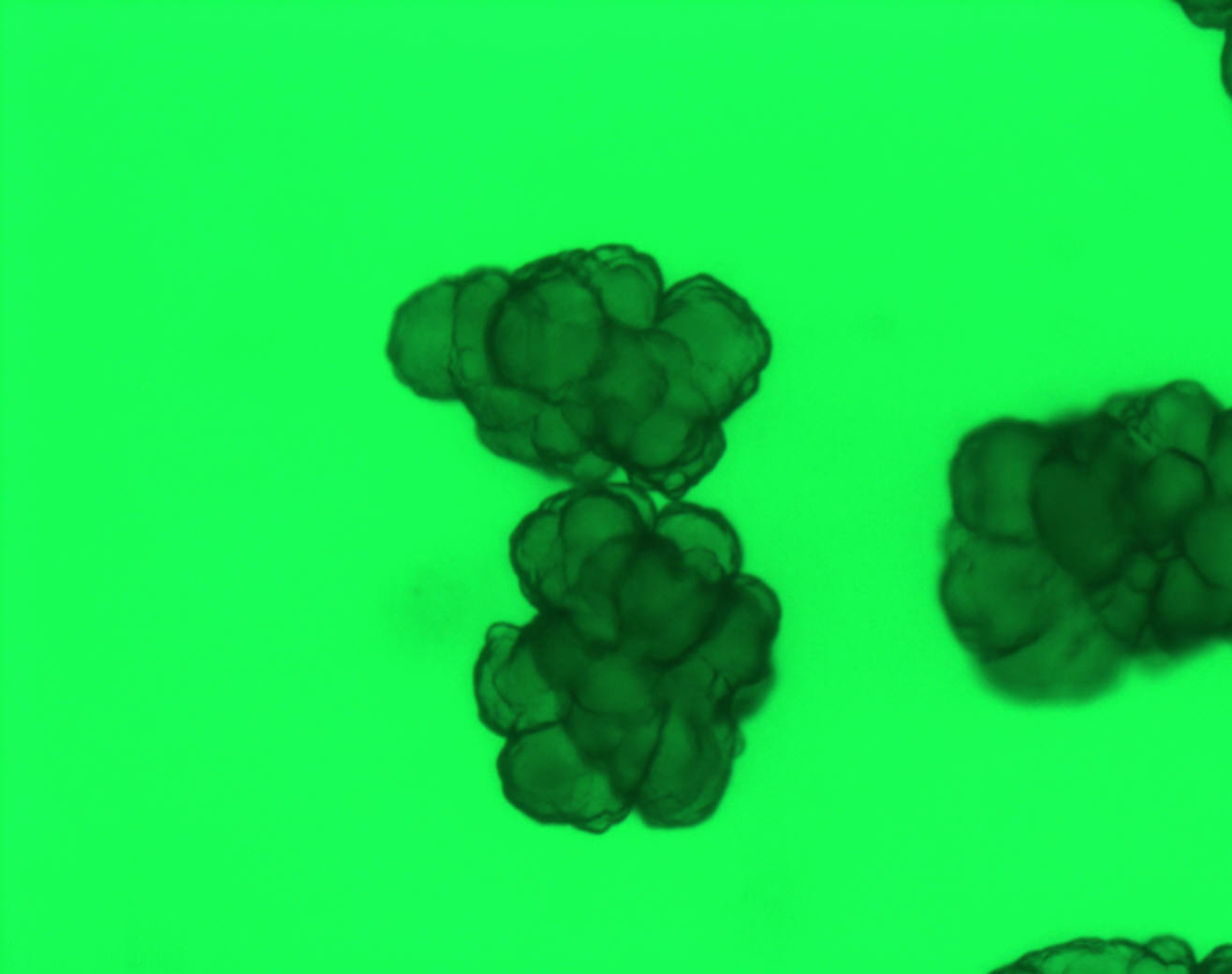

Supplement: Optical photographs [file rsos200663supp4.zip › Figure 5 Optical photographs of PE-120 in LP at 70íμ.jpg]

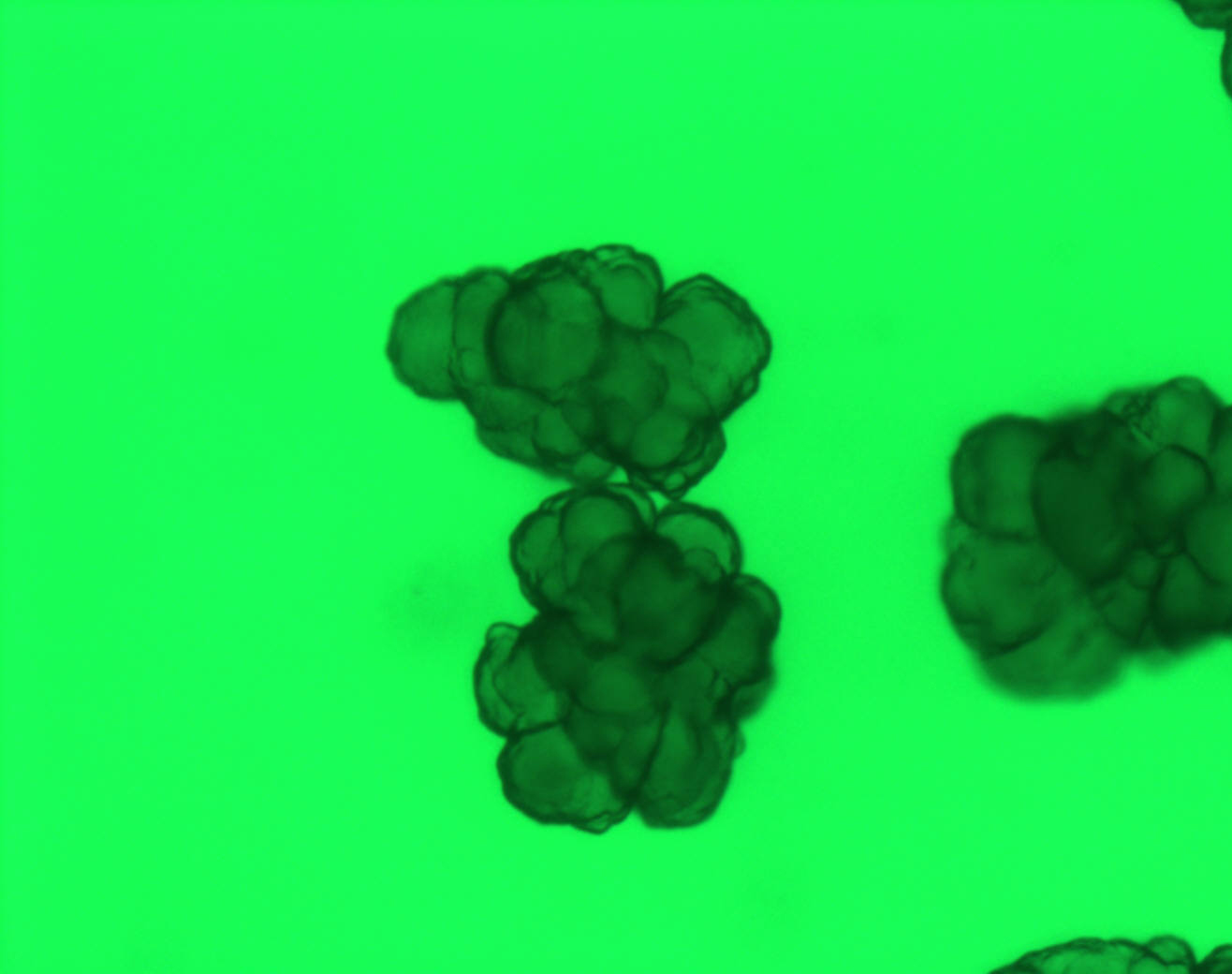

Supplement: Optical photographs [file rsos200663supp4.zip › Figure 5 Optical photographs of PE-120 in LP at90íμ.jpg]

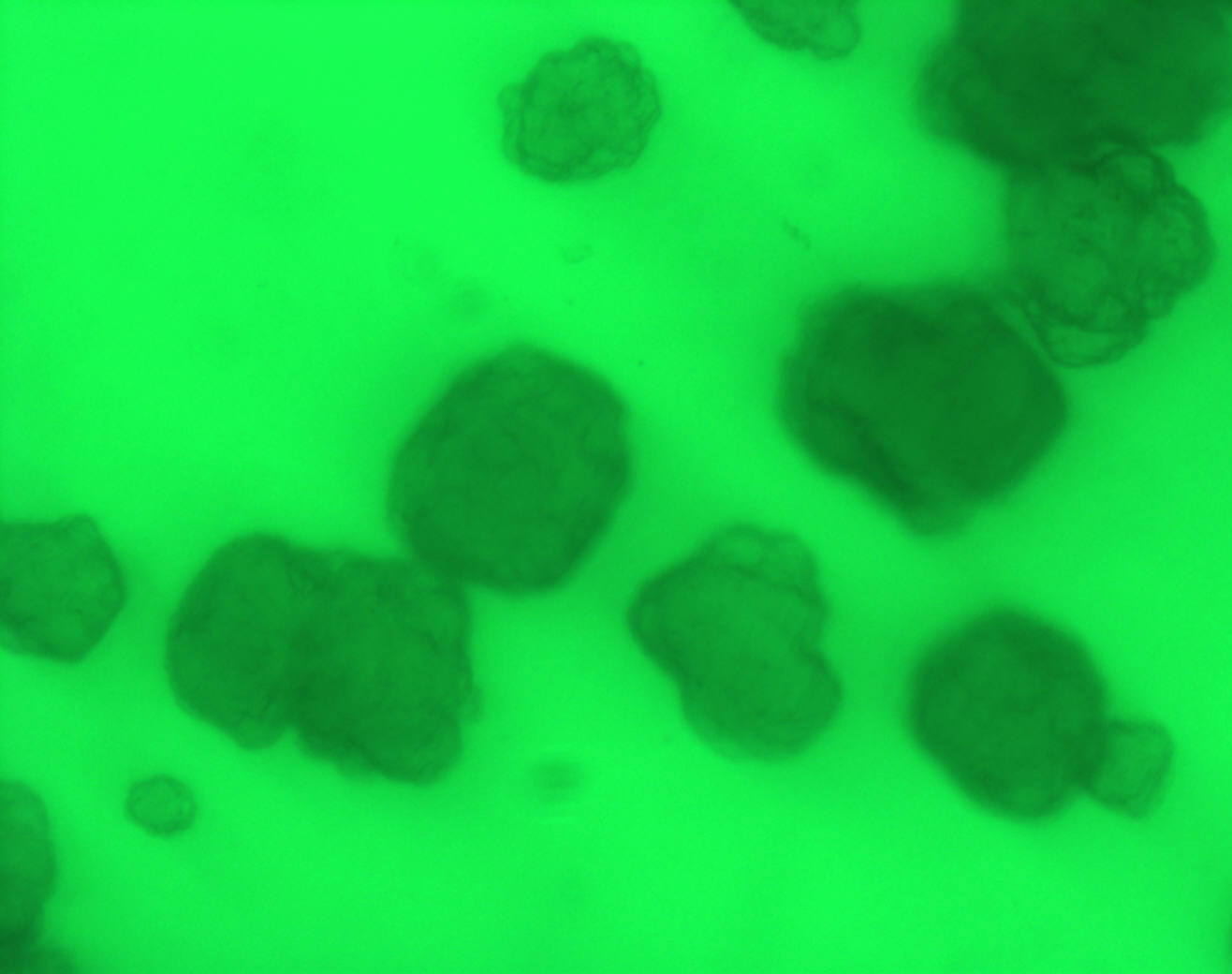

Supplement: Optical photographs [file rsos200663supp4.zip › Figure 6 Optical photographs of PE-140 in LP at 110íμ.jpg]

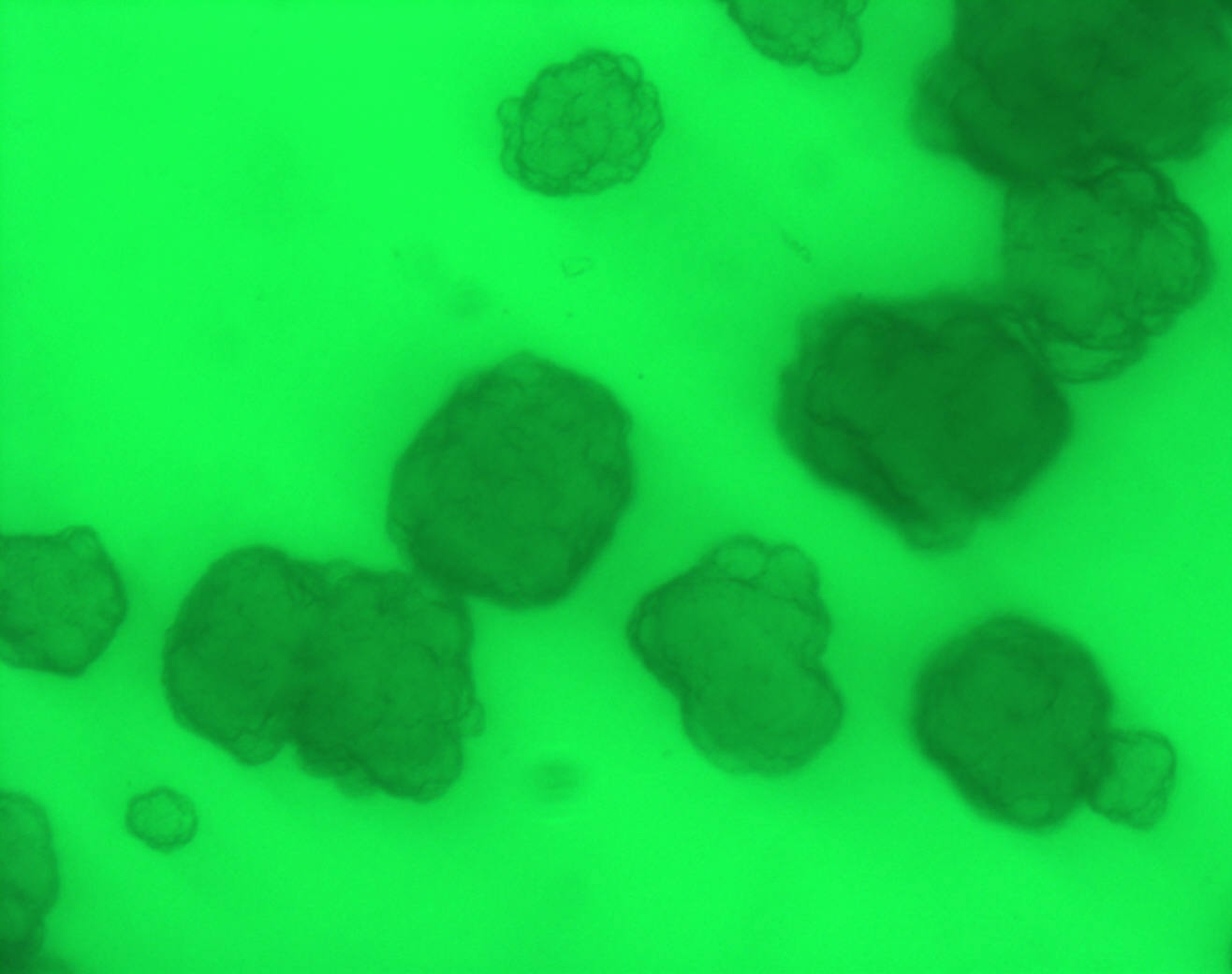

Supplement: Optical photographs [file rsos200663supp4.zip › Figure 6 Optical photographs of PE-140 in LP at 130íμ.jpg]

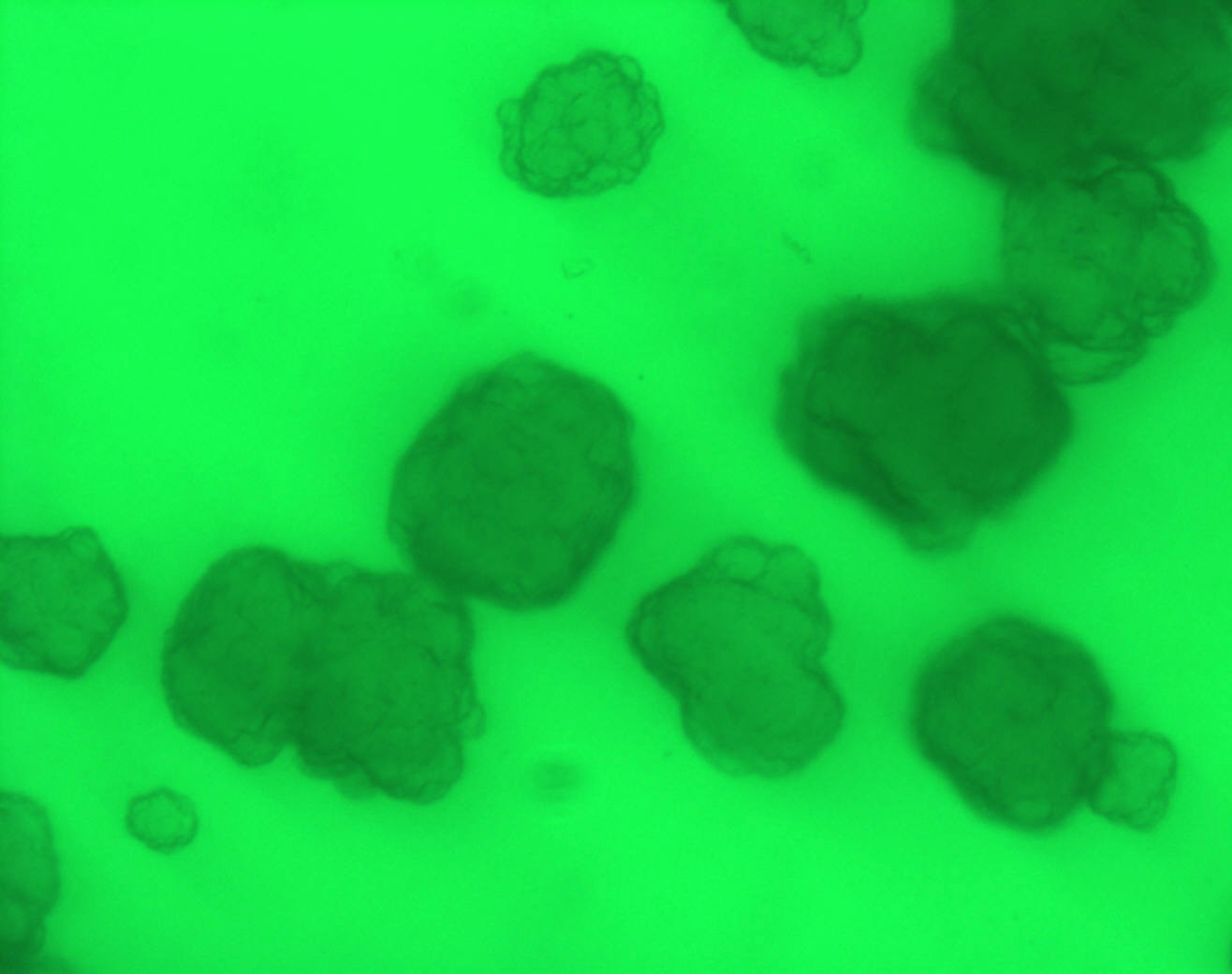

Supplement: Optical photographs [file rsos200663supp4.zip › Figure 6 Optical photographs of PE-140 in LP at 132íμ.jpg]

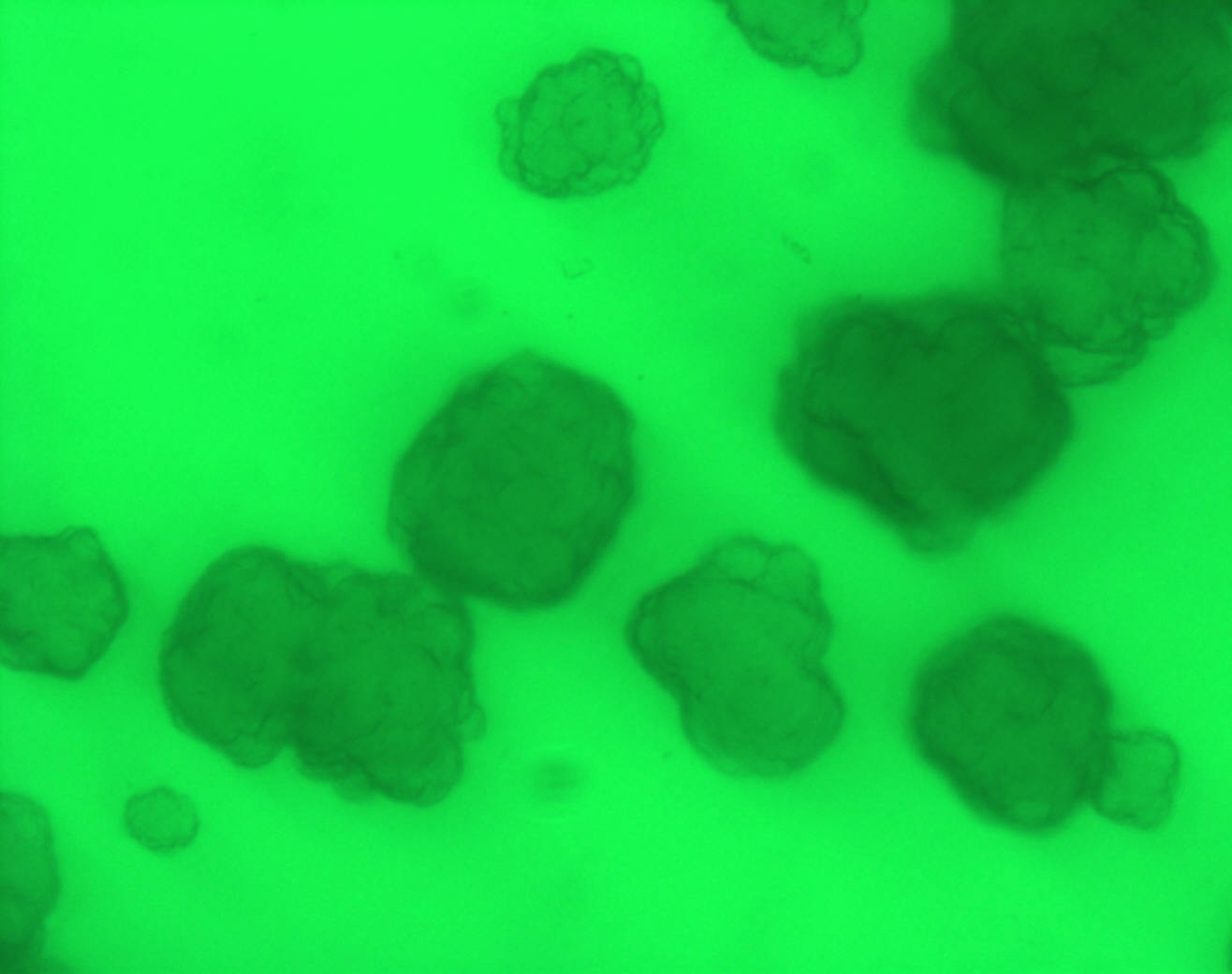

Supplement: Optical photographs [file rsos200663supp4.zip › Figure 6 Optical photographs of PE-140 in LP at 134íμ.jpg]

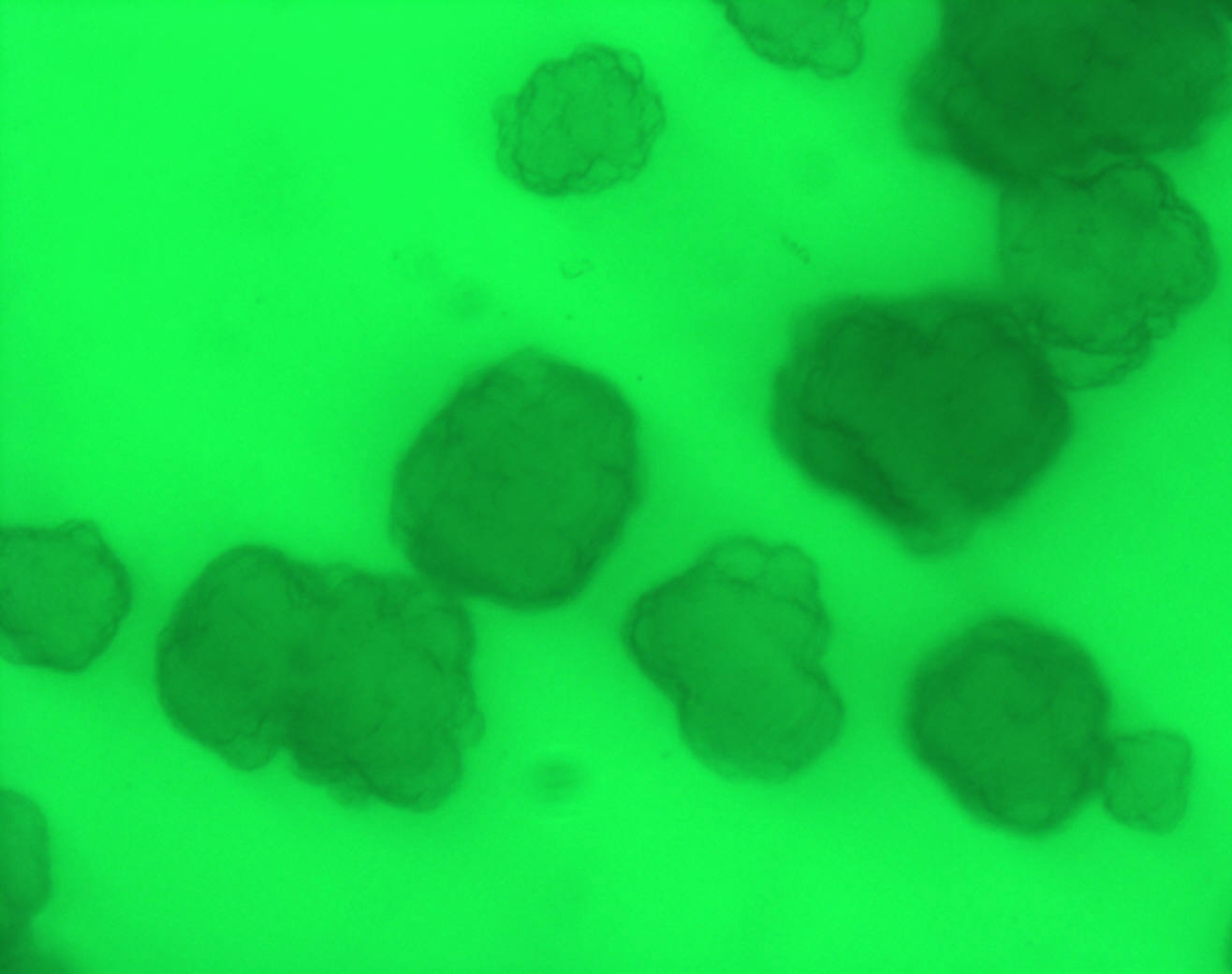

Supplement: Optical photographs [file rsos200663supp4.zip › Figure 6 Optical photographs of PE-140 in LP at 136íμ.jpg]

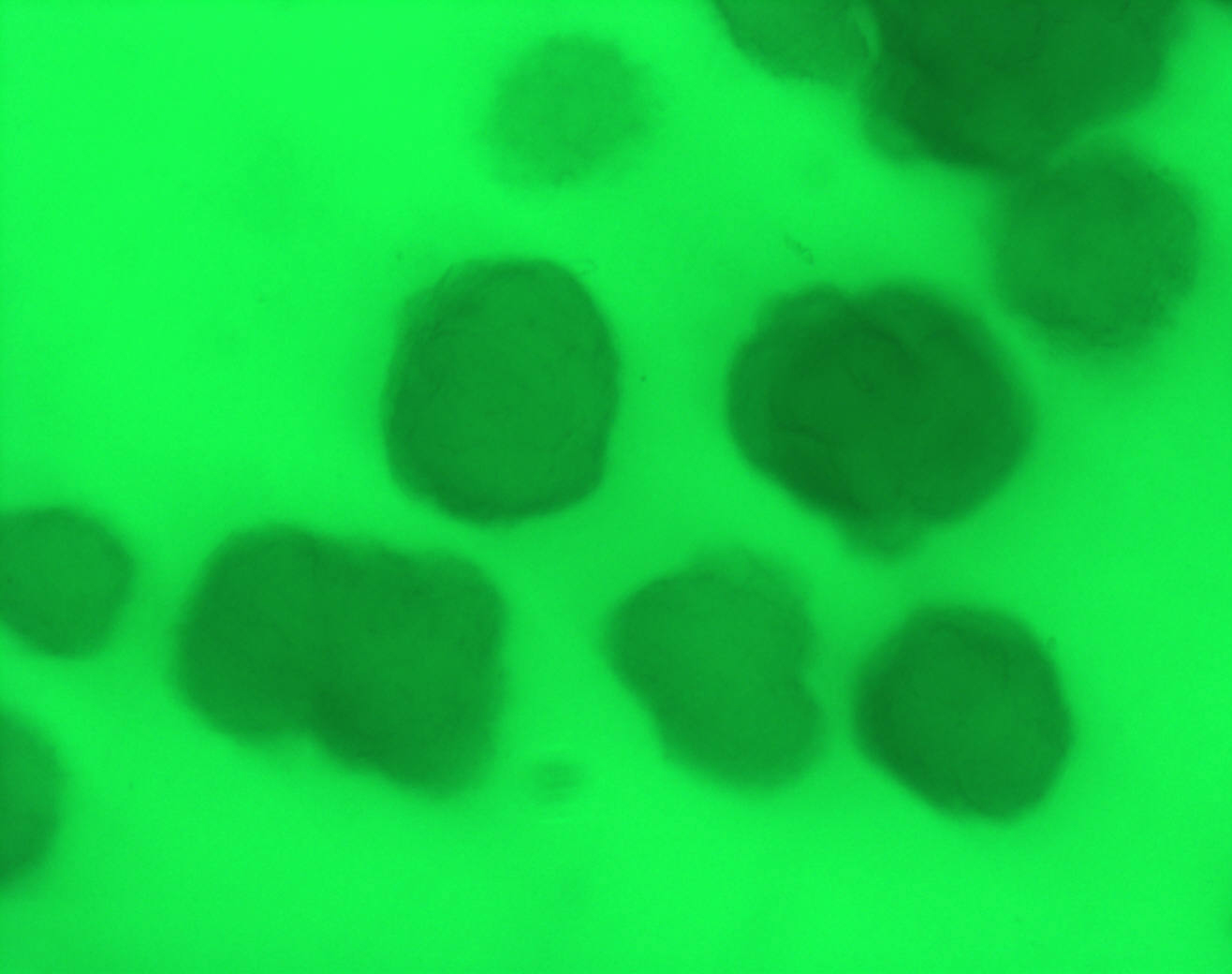

Supplement: Optical photographs [file rsos200663supp4.zip › Figure 6 Optical photographs of PE-140 in LP at 138íμ.jpg]

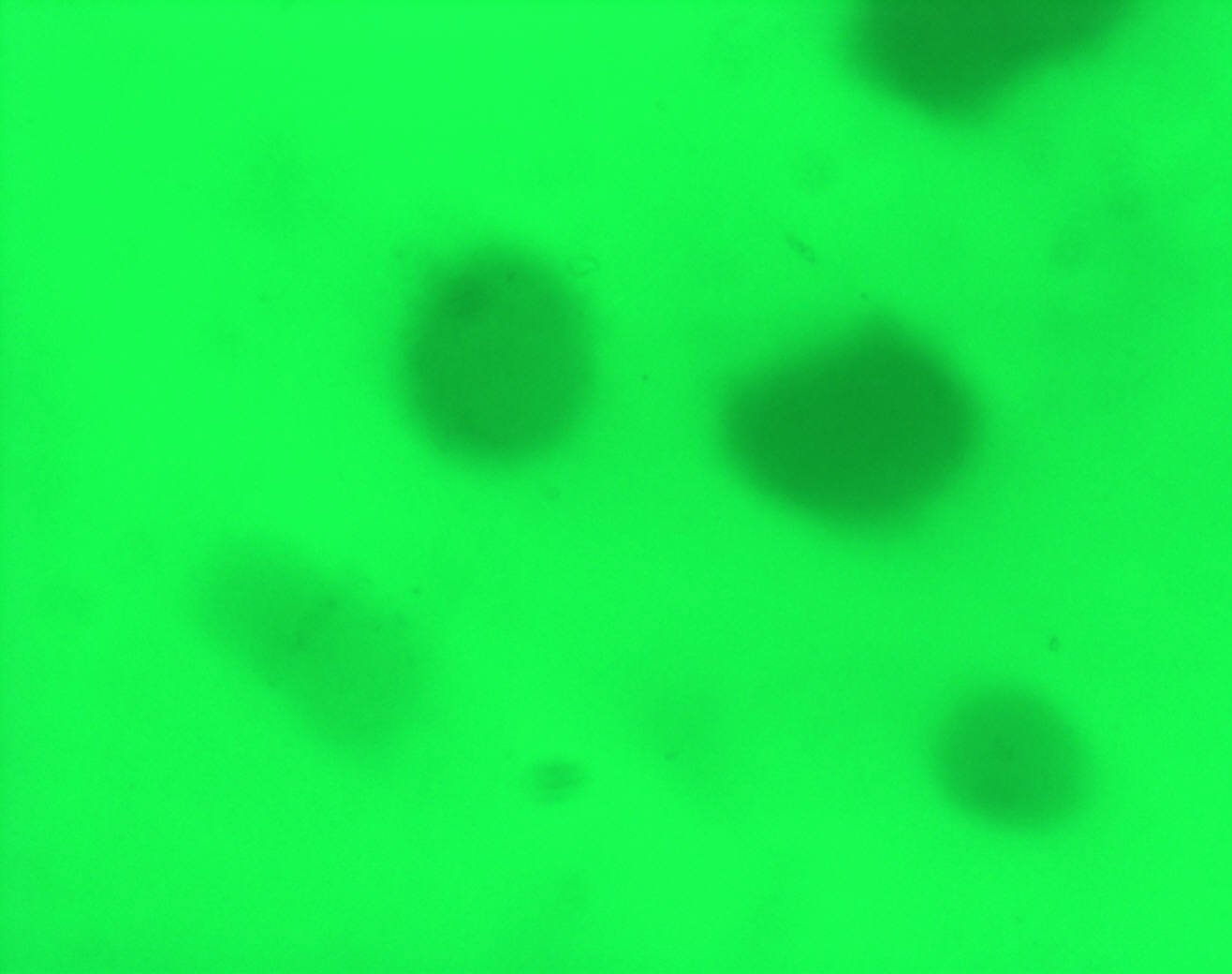

Supplement: Optical photographs [file rsos200663supp4.zip › Figure 6 Optical photographs of PE-140 in LP at 140íμ.jpg]

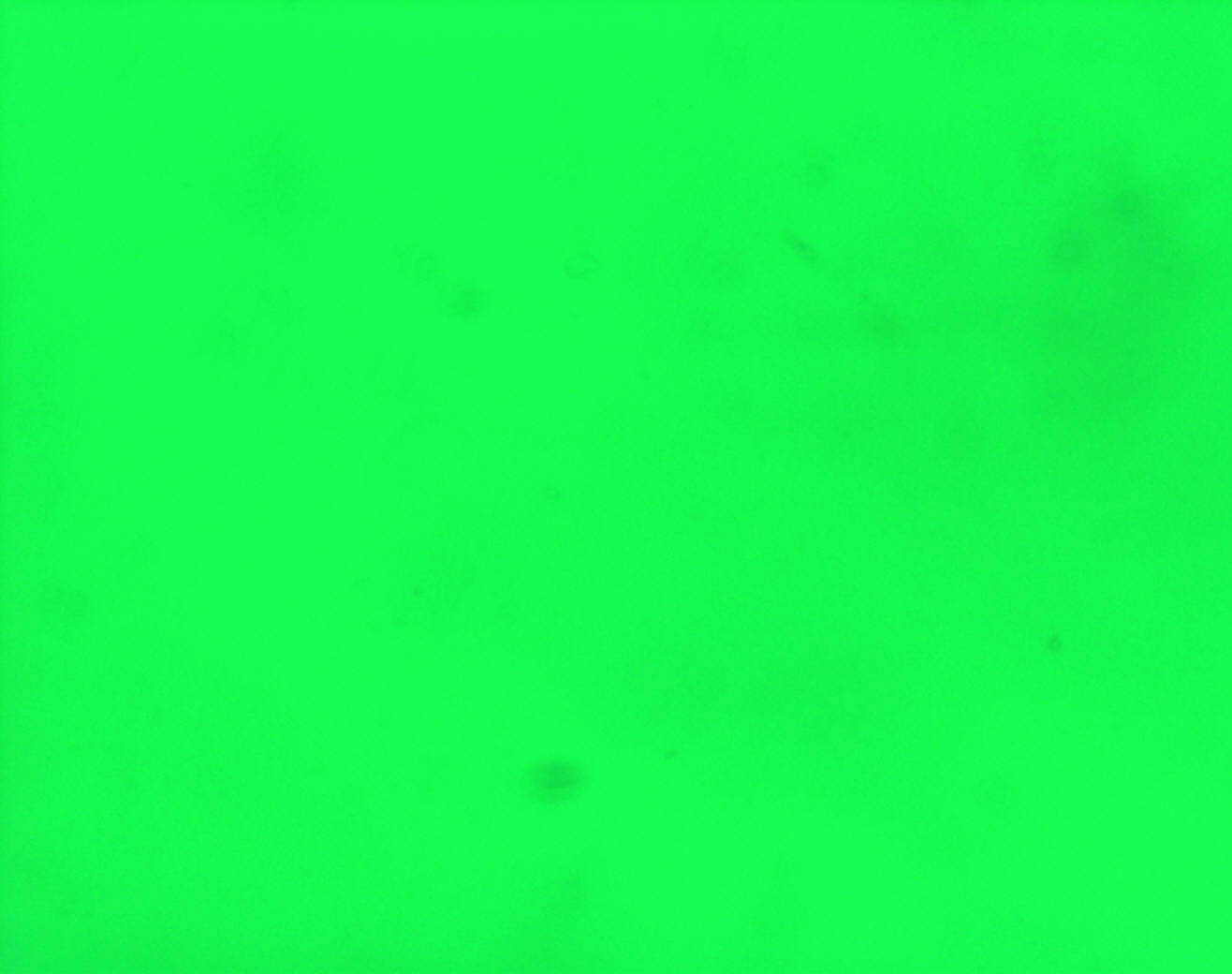

Supplement: Optical photographs [file rsos200663supp4.zip › Figure 6 Optical photographs of PE-140 in LP at 142íμ.jpg]

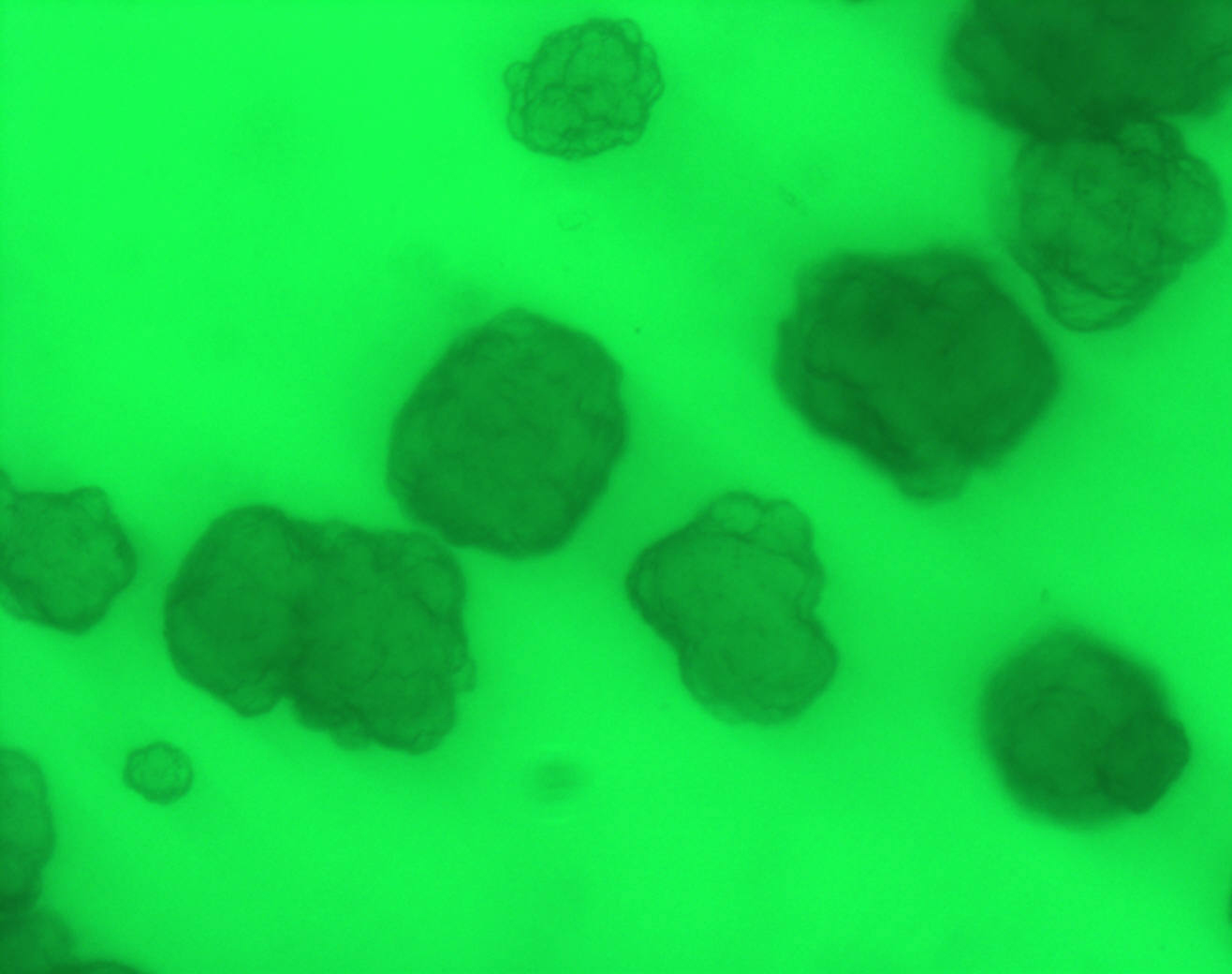

Supplement: Optical photographs [file rsos200663supp4.zip › Figure 6 Optical photographs of PE-140 in LP at 30íμ.jpg]

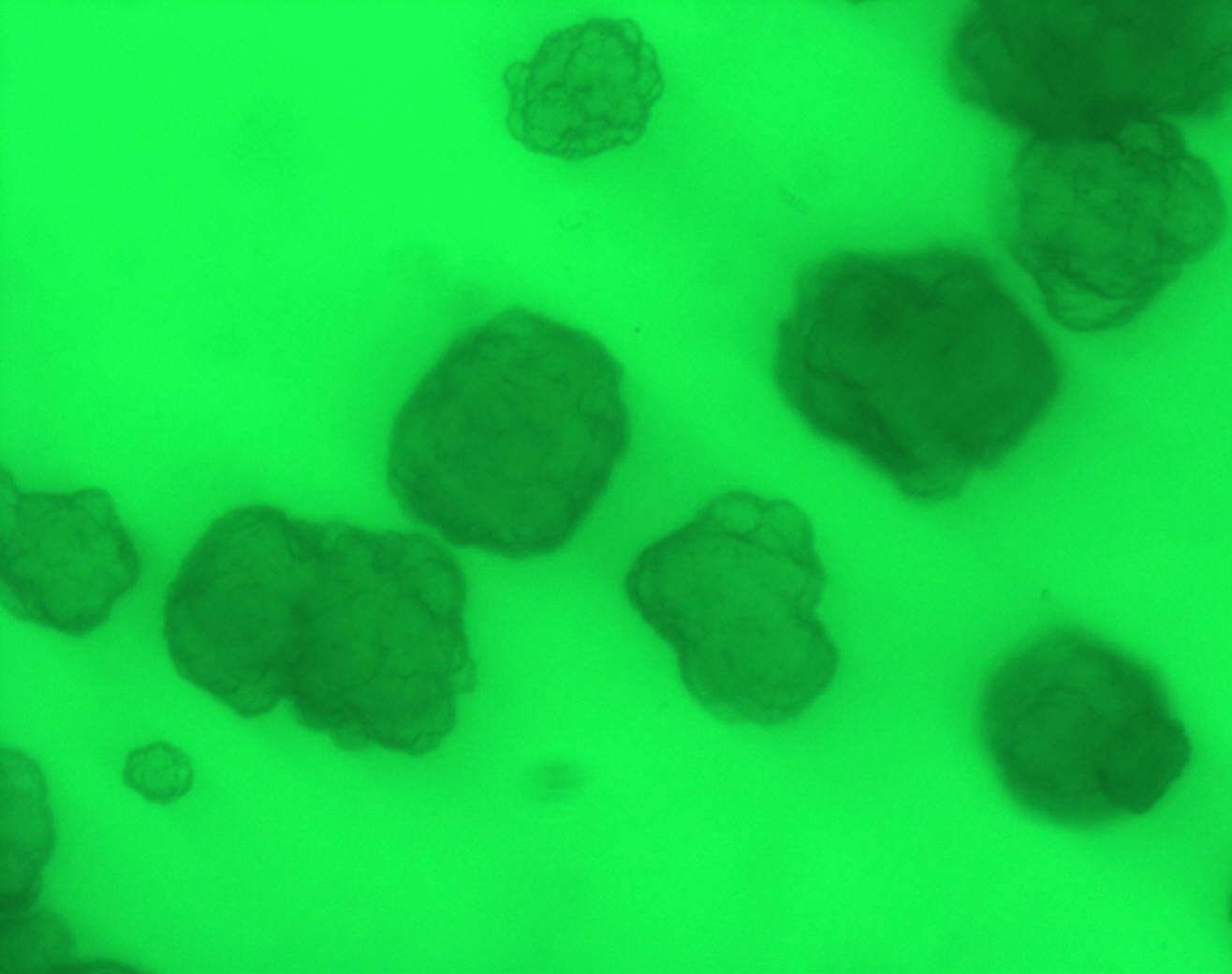

Supplement: Optical photographs [file rsos200663supp4.zip › Figure 6 Optical photographs of PE-140 in LP at 50íμ.jpg]

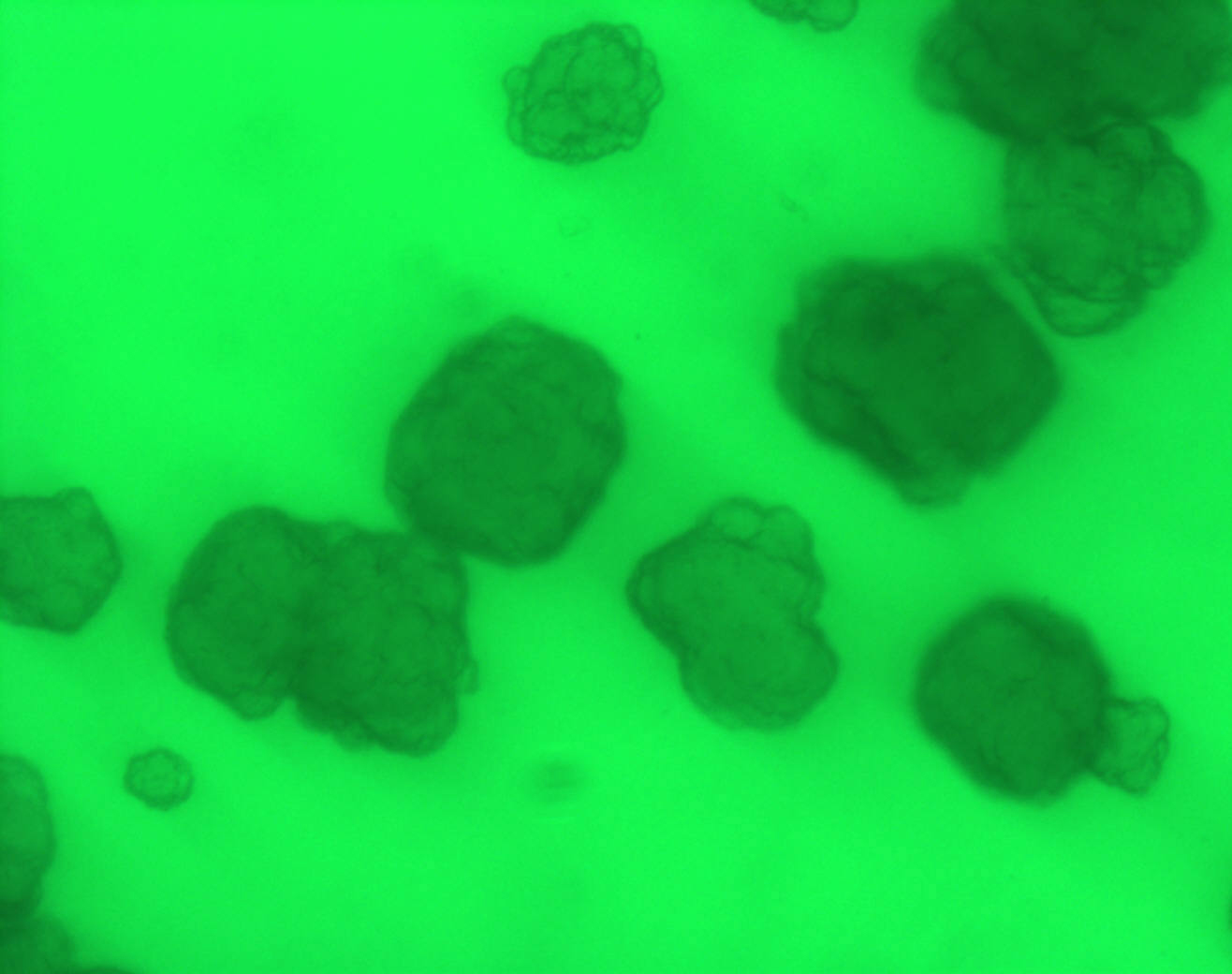

Supplement: Optical photographs [file rsos200663supp4.zip › Figure 6 Optical photographs of PE-140 in LP at 70íμ.jpg]

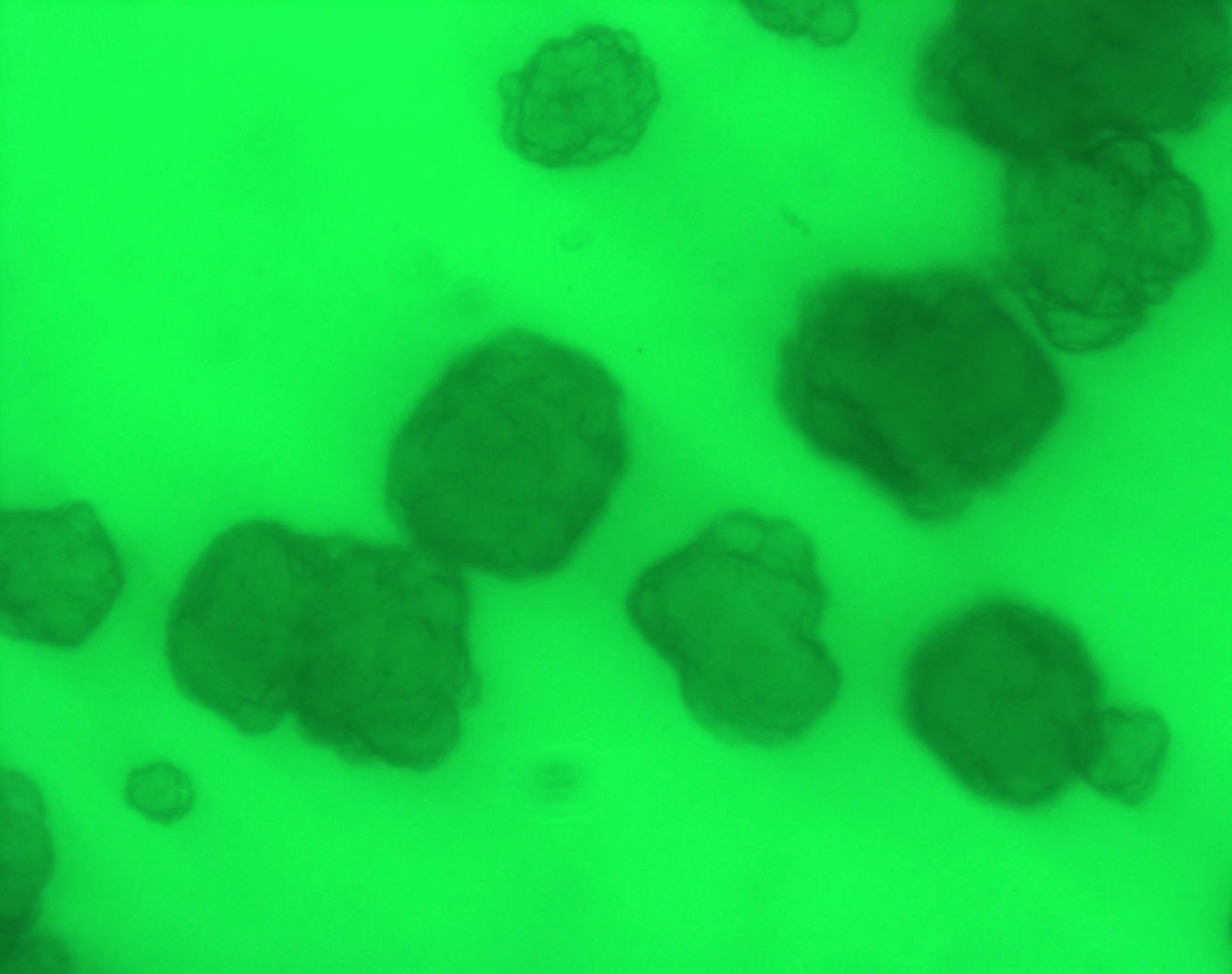

Supplement: Optical photographs [file rsos200663supp4.zip › Figure 6 Optical photographs of PE-140 in LP at 90íμ.jpg]

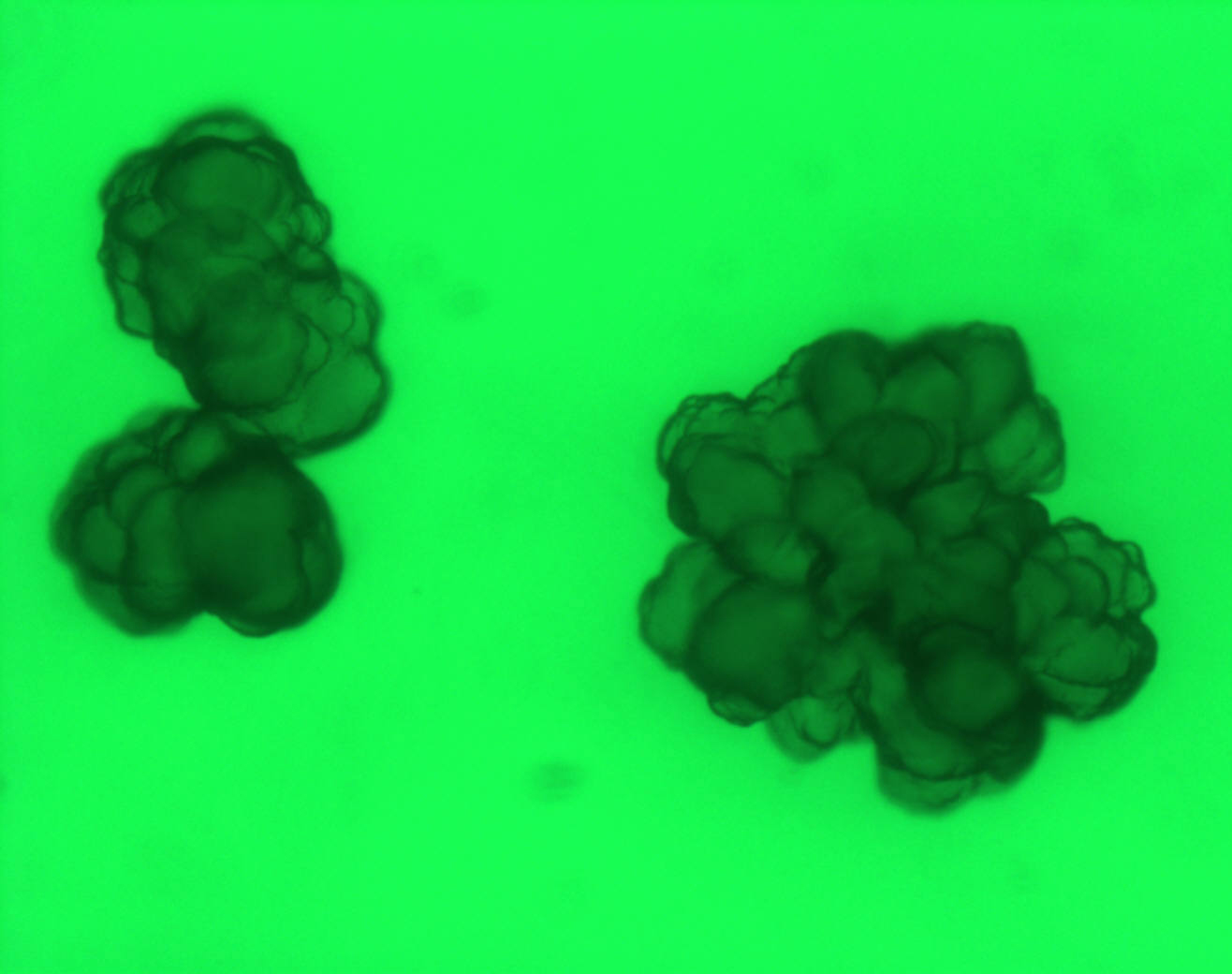

Supplement: Optical photographs [file rsos200663supp4.zip › Figure 7 Optical photographs of PE-120 in LP at 110íμ.jpg]

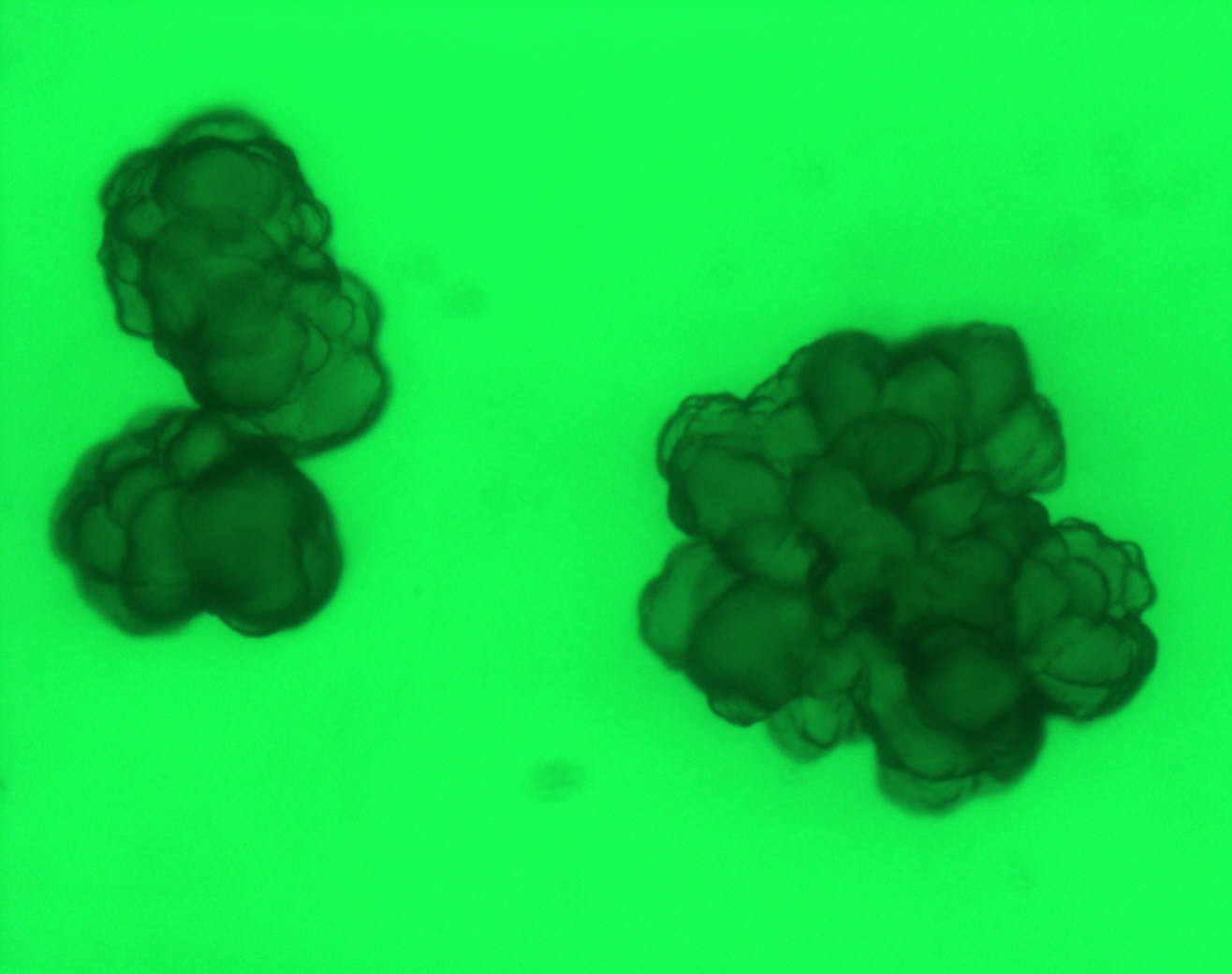

Supplement: Optical photographs [file rsos200663supp4.zip › Figure 7 Optical photographs of PE-120 in LP at 130íμ.jpg]

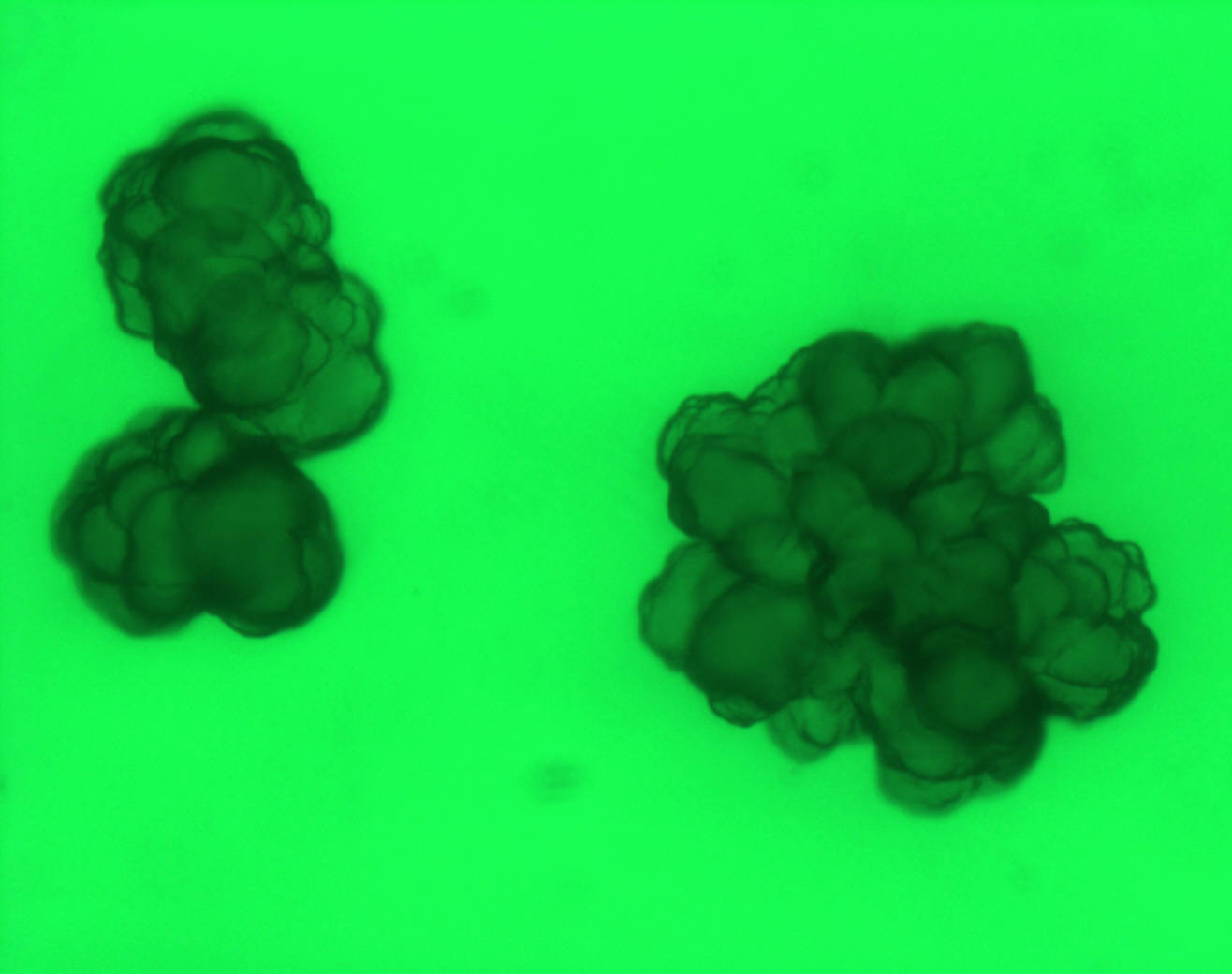

Supplement: Optical photographs [file rsos200663supp4.zip › Figure 7 Optical photographs of PE-120 in LP at 132íμ.jpg]

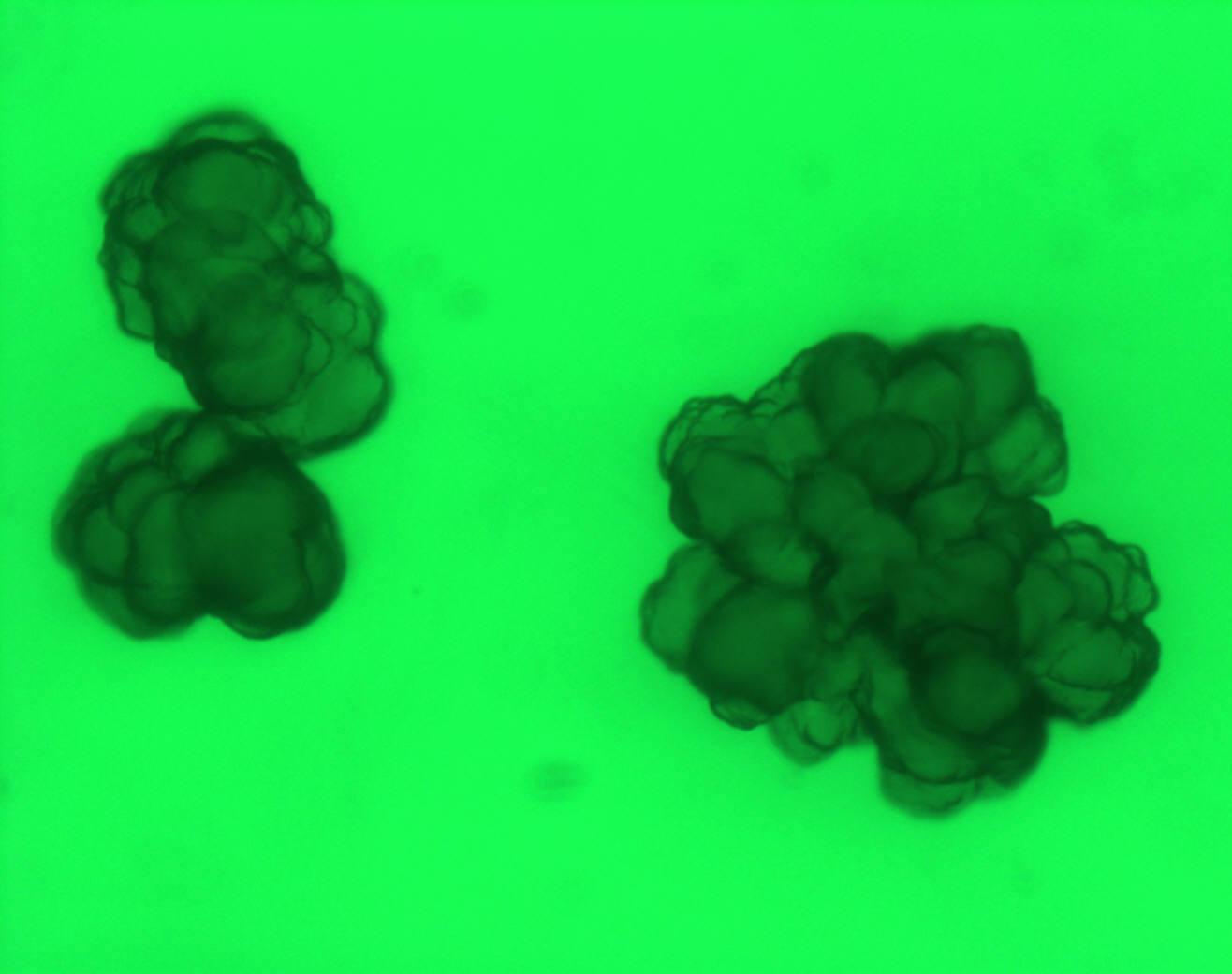

Supplement: Optical photographs [file rsos200663supp4.zip › Figure 7 Optical photographs of PE-120 in LP at 134íμ.jpg]

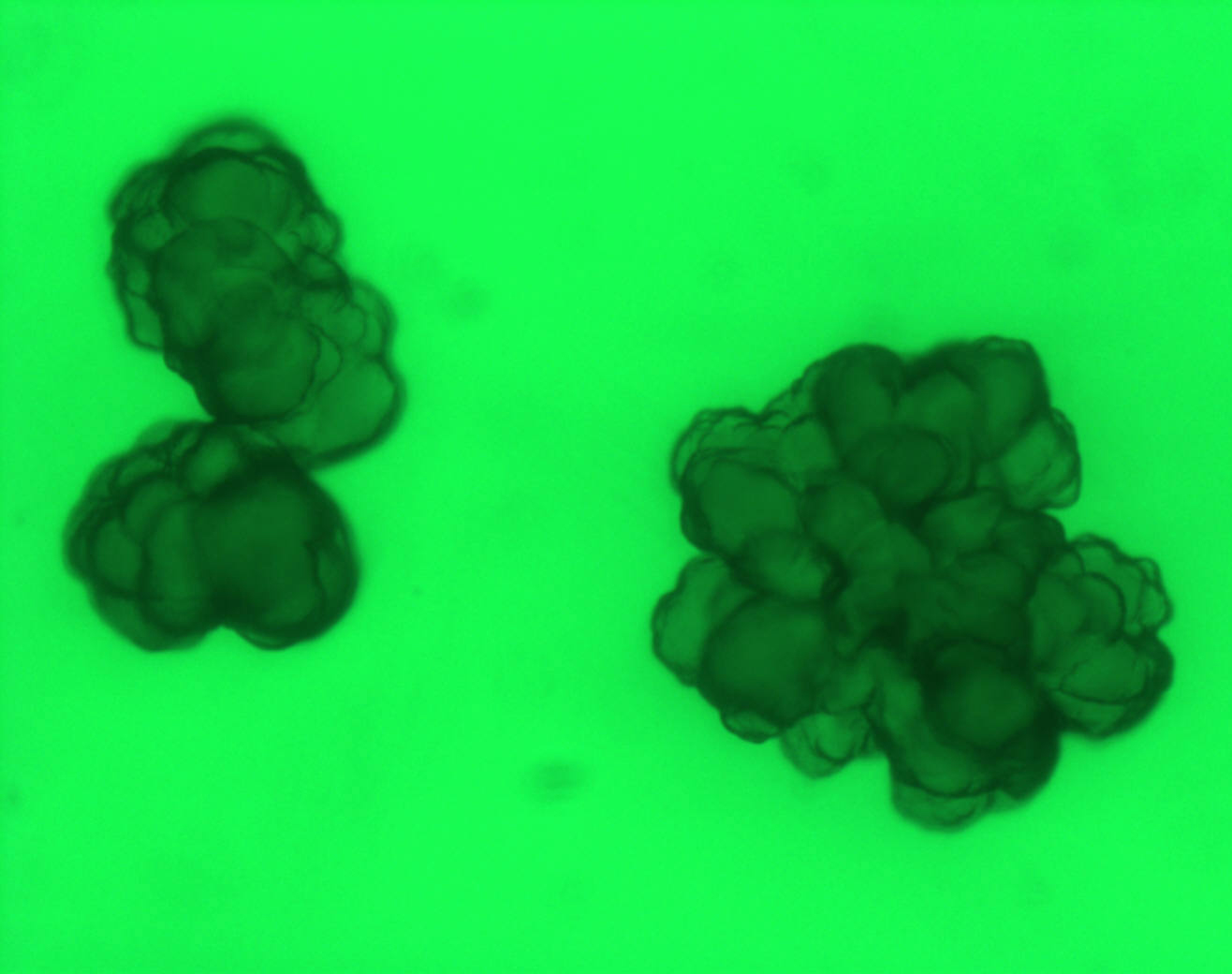

Supplement: Optical photographs [file rsos200663supp4.zip › Figure 7 Optical photographs of PE-120 in LP at 136íμ.jpg]

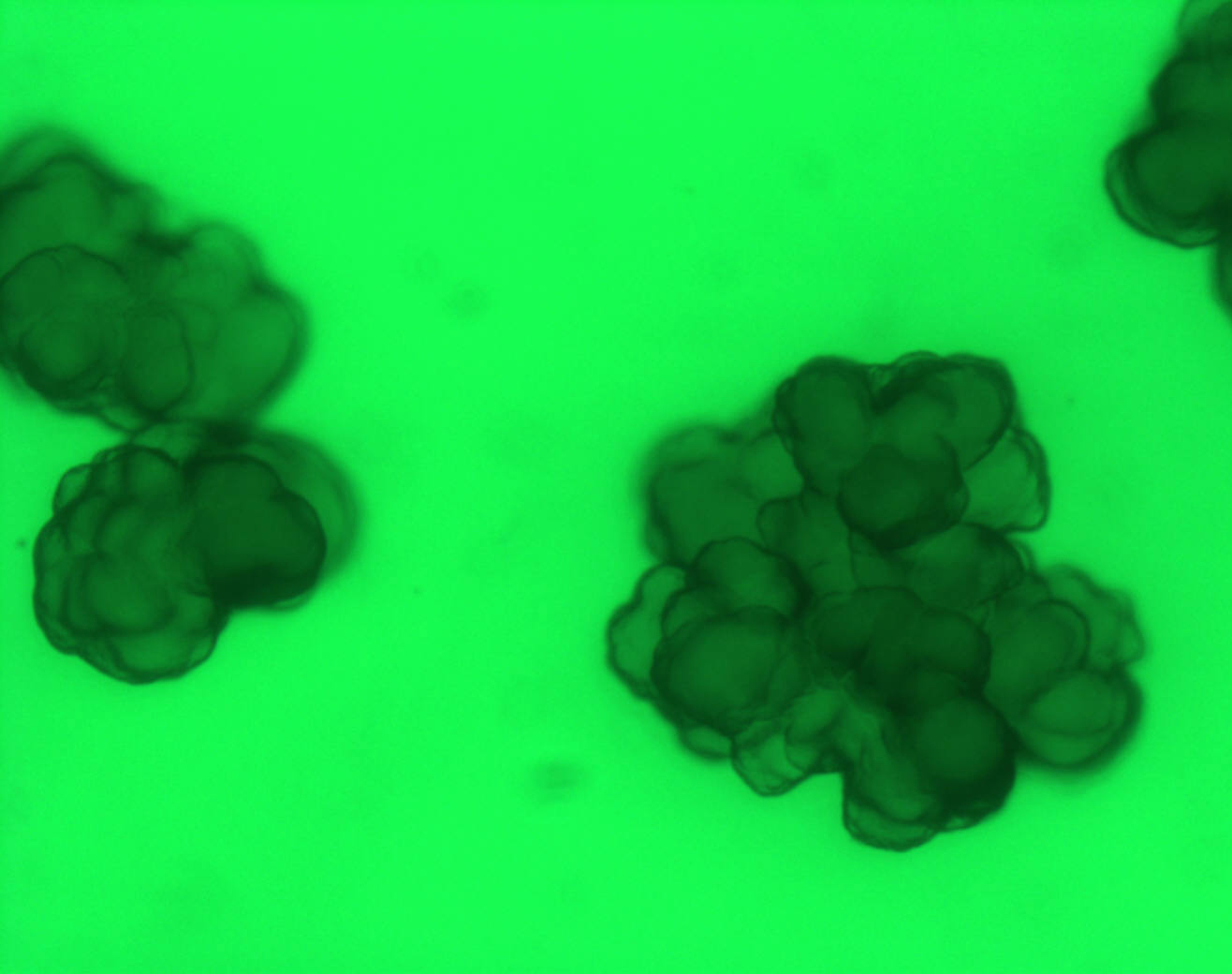

Supplement: Optical photographs [file rsos200663supp4.zip › Figure 7 Optical photographs of PE-120 in LP at 138íμ.jpg]

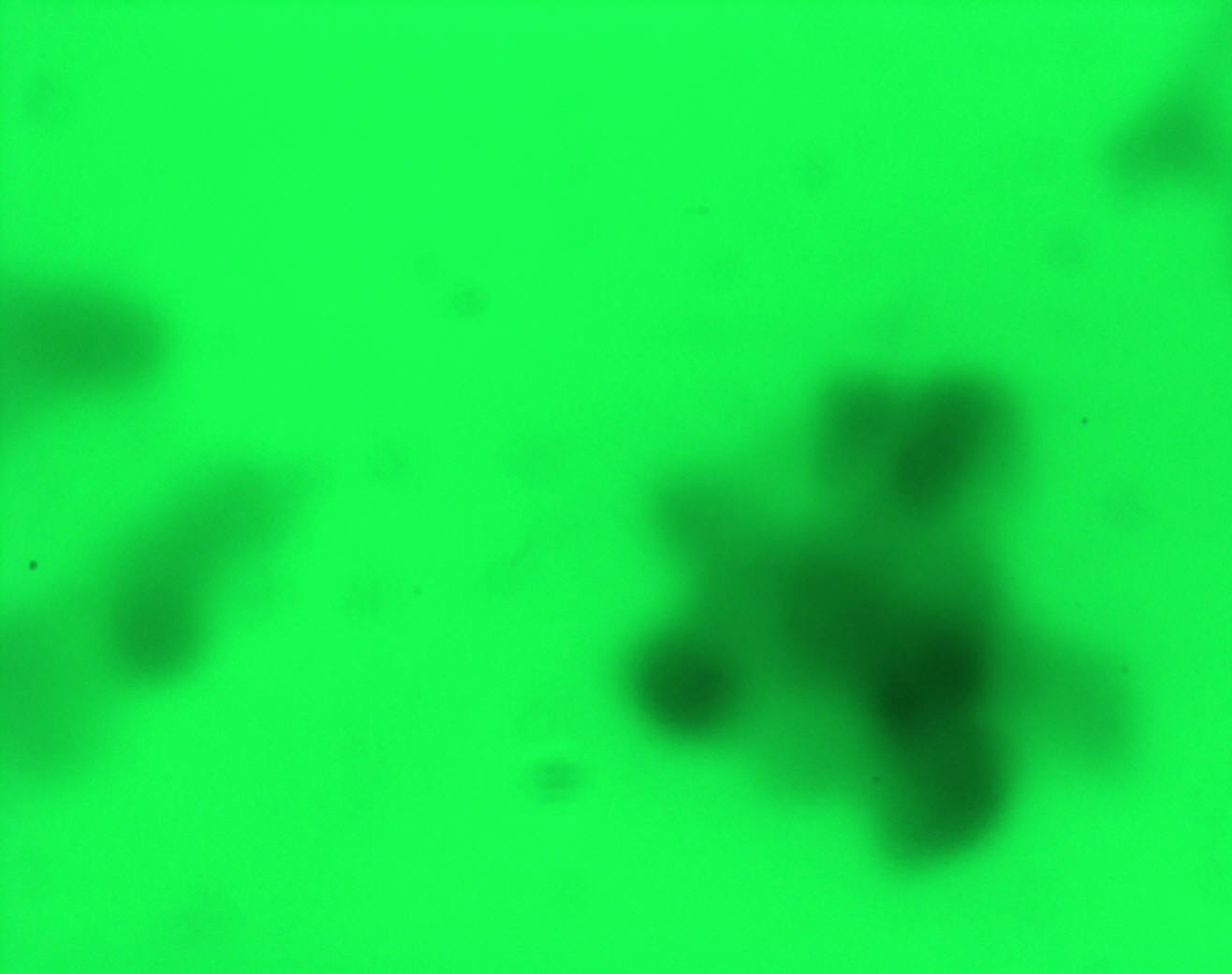

Supplement: Optical photographs [file rsos200663supp4.zip › Figure 7 Optical photographs of PE-120 in LP at 140íμ.jpg]

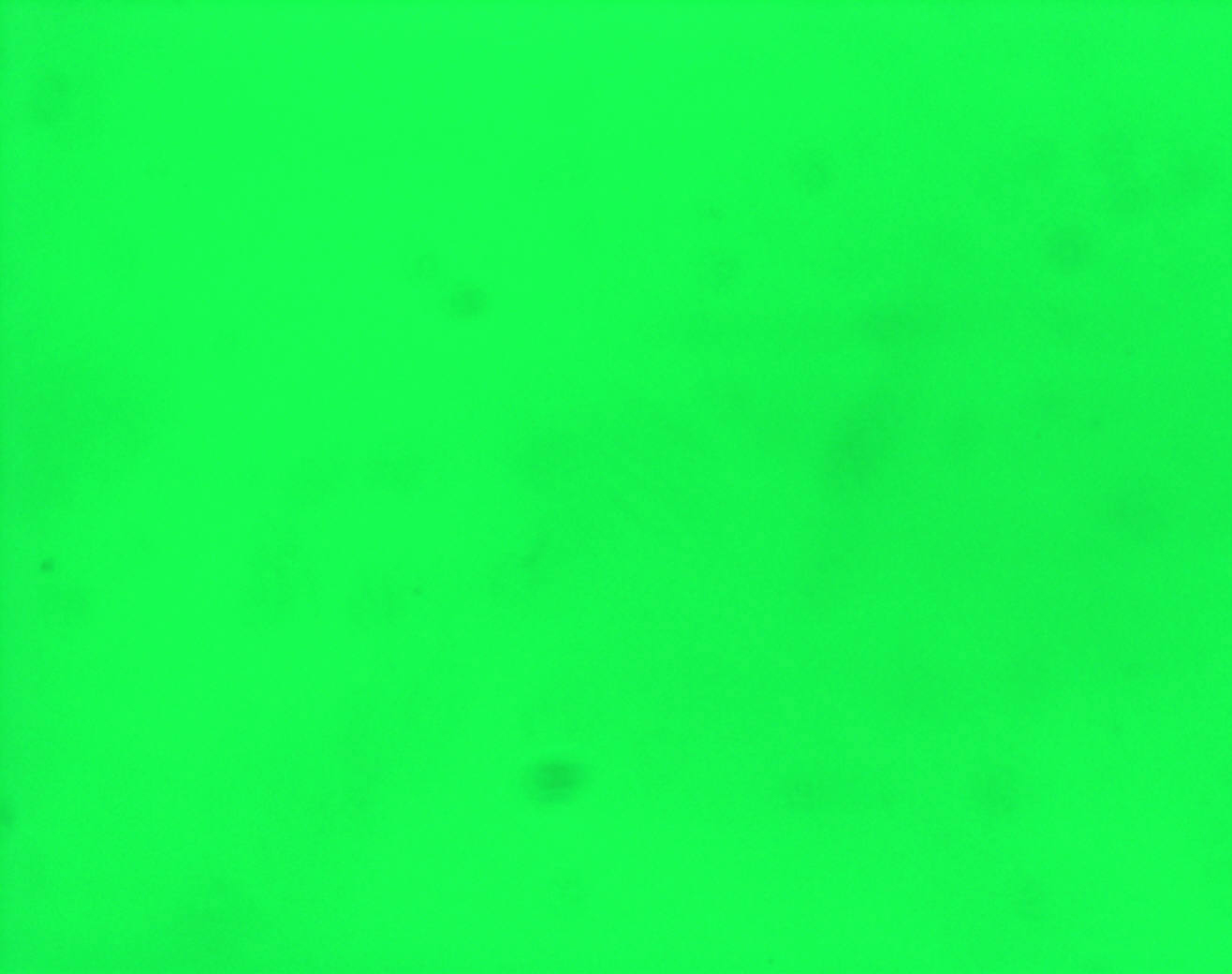

Supplement: Optical photographs [file rsos200663supp4.zip › Figure 7 Optical photographs of PE-120 in LP at 142íμ.jpg]

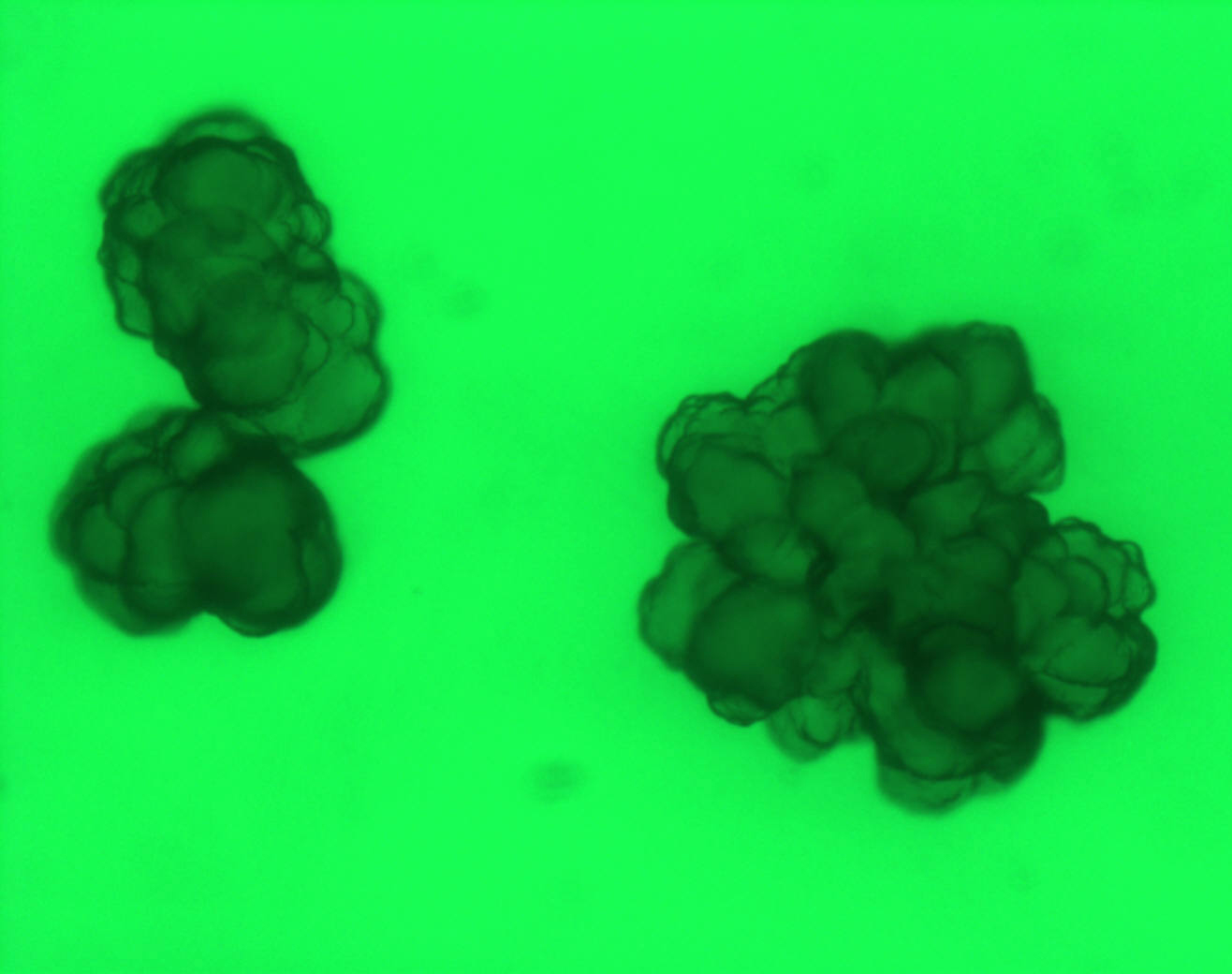

Supplement: Optical photographs [file rsos200663supp4.zip › Figure 7 Optical photographs of PE-120 in LP at 30íμ.jpg]

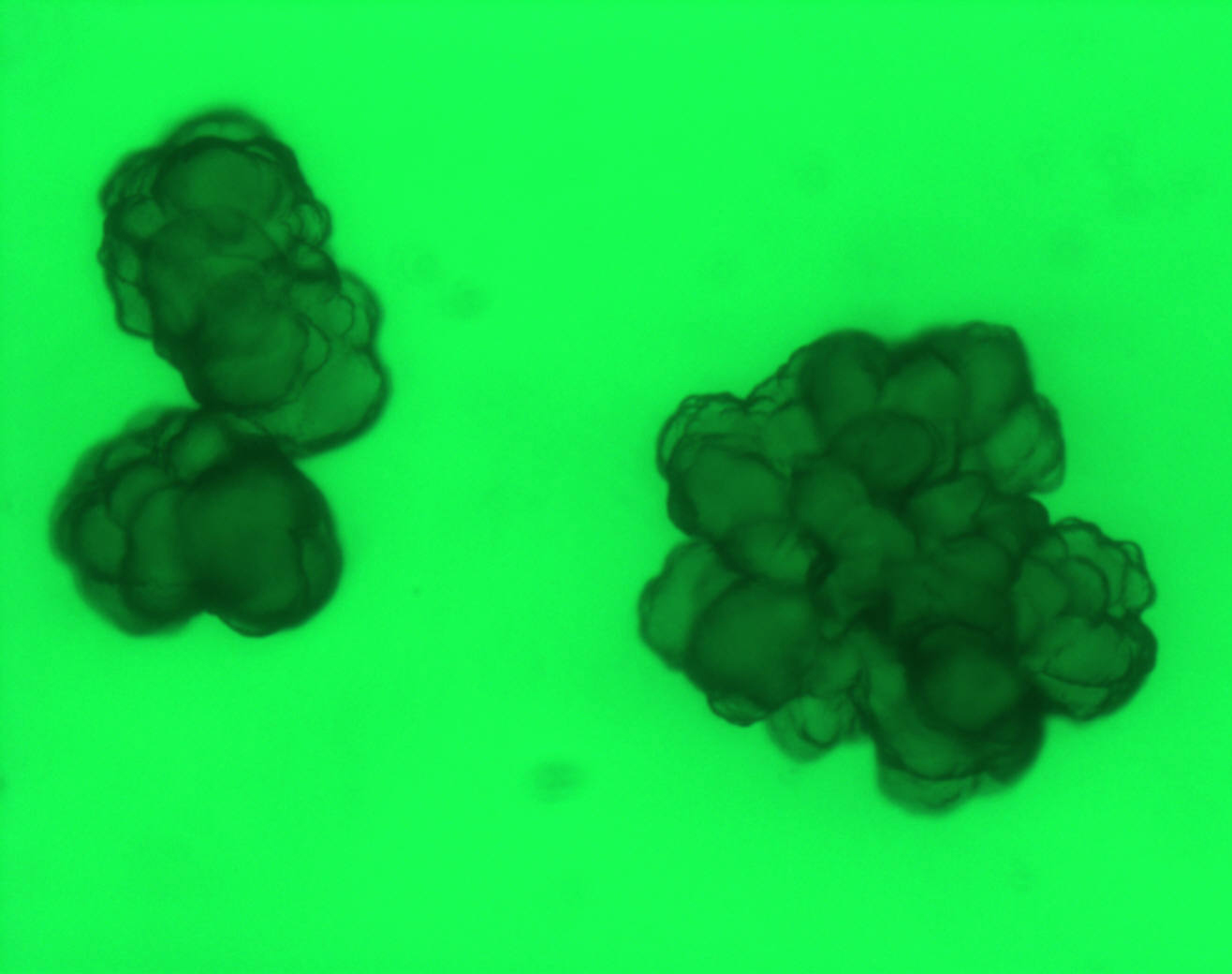

Supplement: Optical photographs [file rsos200663supp4.zip › Figure 7 Optical photographs of PE-120 in LP at 50íμ.jpg]

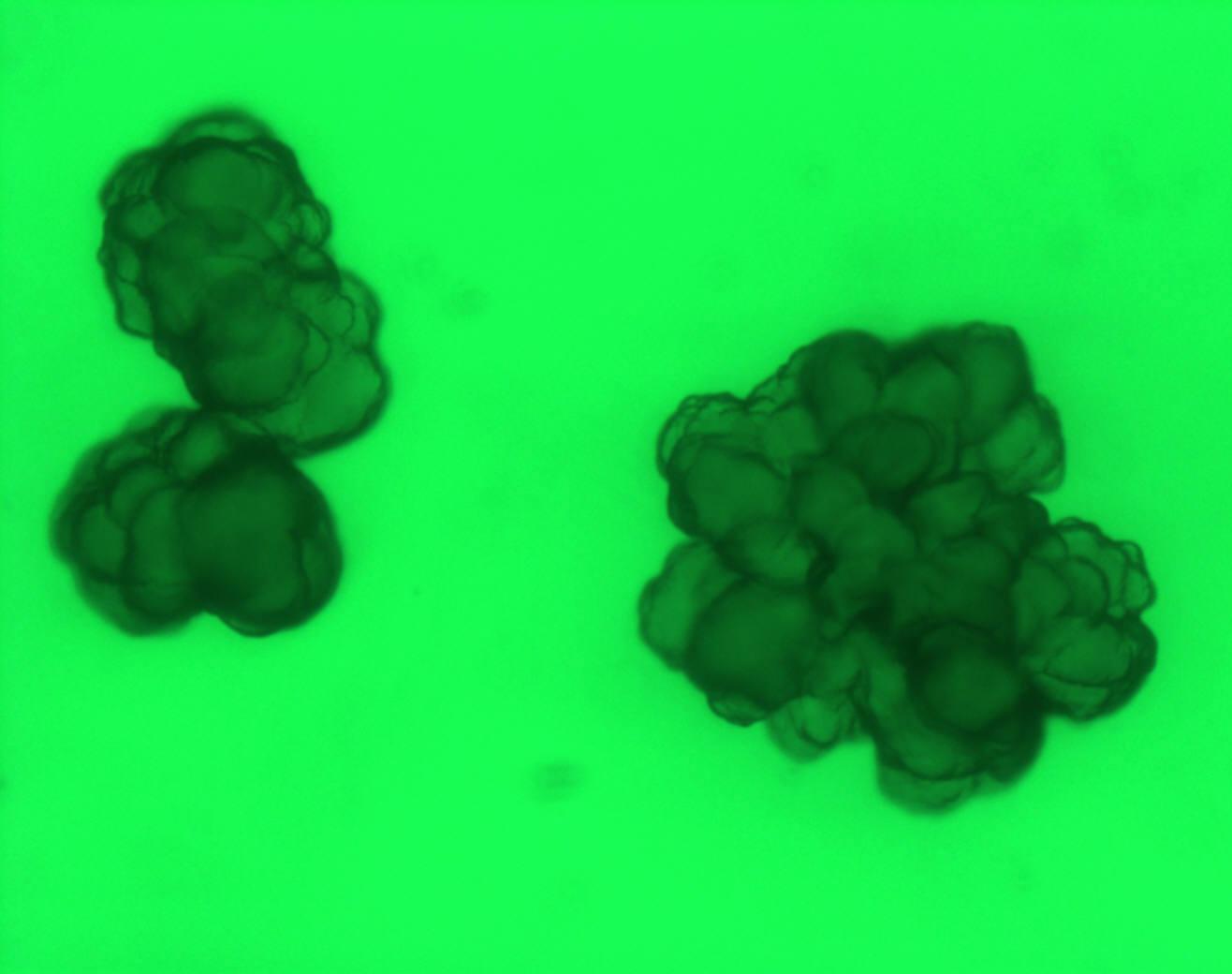

Supplement: Optical photographs [file rsos200663supp4.zip › Figure 7 Optical photographs of PE-120 in LP at 70íμ.jpg]

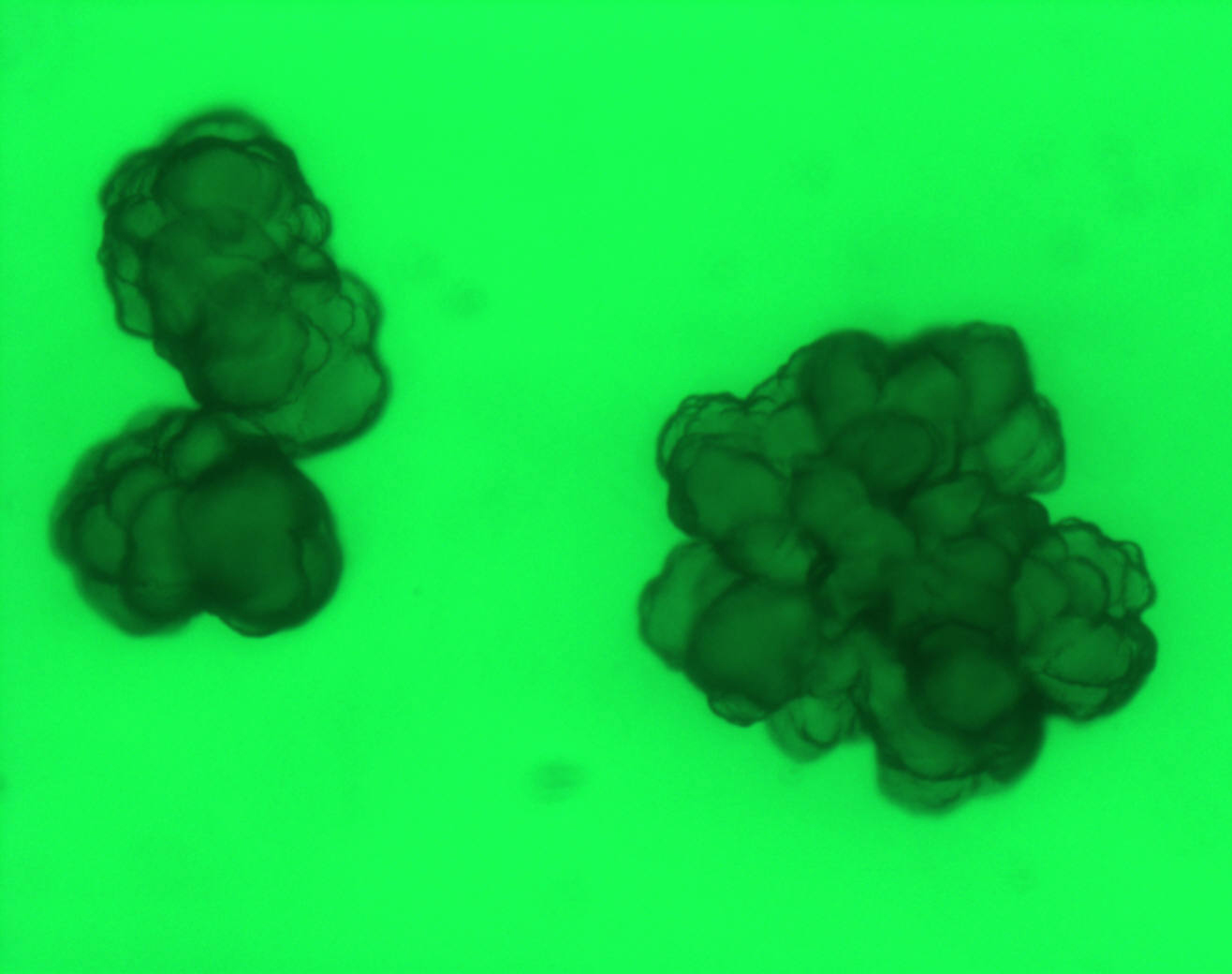

Supplement: Optical photographs [file rsos200663supp4.zip › Figure 7 Optical photographs of PE-120 in LP at 90íμ.jpg]
